# Thorough Investigation of the Phenolic Profile of Reputable Greek Honey Varieties: Varietal Discrimination and Floral Markers Identification Using Liquid Chromatography–High-Resolution Mass Spectrometry

**DOI:** 10.3390/molecules27144444

**Published:** 2022-07-11

**Authors:** Georgios A. Koulis, Aristeidis S. Tsagkaris, Panagiota A. Katsianou, Panagiotis-Loukas P. Gialouris, Ioannis Martakos, Fotis Stergiou, Alberto Fiore, Eleni I. Panagopoulou, Sofia Karabournioti, Carsten Baessmann, Noud van der Borg, Marilena E. Dasenaki, Charalampos Proestos, Nikolaos S. Thomaidis

**Affiliations:** 1 Analytical Chemistry Laboratory, Chemistry Department, National and Kapodistrian University of Athens, Panepistimiopolis Zographou, 15771 Athens, Greece; georgekoulis@chem.uoa.gr (G.A.K.); katsianp@chem.uoa.gr (P.A.K.); pgialour@chem.uoa.gr (P.-L.P.G.); johnmrtk@chem.uoa.gr (I.M.); fotis.stergiou@outlook.com (F.S.); elenapanag@chem.uoa.gr (E.I.P.); 2 Food Chemistry Laboratory, Department of Chemistry, National and Kapodistrian University of Athens, Panepistimiopolis Zographou, 15771 Athens, Greece; harpro@chem.uoa.gr; 3 Department of Food Analysis and Nutrition, Faculty of Food and Biochemical Technology, University of Chemistry and Technology Prague, Technická 5, 16628 Prague, Czech Republic; tsagkara@vscht.cz; 4 Division of Engineering and Food Science, School of Applied Science, Abertay University, Bell Street, Dundee DD1 1HG, UK; a.fiore@abertay.ac.uk; 5 Attiki Honey SA, Kryoneri Attikis, 14568 Athens, Greece; skar@attiki-pittas.gr; 6 Bruker Daltonik GmbH, Fahrenheitstraße 4, 28359 Bremen, Germany; carsten.baessmann@bruker.com (C.B.); noud.van_der_borg@bruker.com (N.v.d.B.)

**Keywords:** honey, phenolic compounds, metabolomics, Greek honey, chemometrics, botanical origin, authenticity, discrimination, high-resolution mass spectrometry

## Abstract

Honey is a highly consumed commodity due to its potential health benefits upon certain consumption, resulting in a high market price. This fact indicates the need to protect honey from fraudulent acts by delivering comprehensive analytical methodologies. In this study, targeted, suspect and non-targeted metabolomic workflows were applied to identify botanical origin markers of Greek honey. Blossom honey samples (*n* = 62) and the unifloral fir (*n* = 10), oak (*n* = 24), pine (*n* = 39) and thyme (*n* = 34) honeys were analyzed using an ultra-high-performance liquid chromatography hybrid quadrupole time-of-flight mass spectrometry (UHPLC-q-TOF-MS) system. Several potential authenticity markers were revealed from the application of different metabolomic workflows. In detail, based on quantitative targeted analysis, three blossom honey markers were found, namely, galangin, pinocembrin and chrysin, while gallic acid concentration was found to be significantly higher in oak honey. Using suspect screening workflow, 12 additional bioactive compounds were identified and semi-quantified, achieving comprehensive metabolomic honey characterization. Lastly, by combining non-targeted screening with advanced chemometrics, it was possible to discriminate thyme from blossom honey and develop binary discriminatory models with high predictive power. In conclusion, a holistic approach to assessing the botanical origin of Greek honey is presented, highlighting the complementarity of the three applied metabolomic approaches.

## 1. Introduction

Honey is a natural sweetener widely consumed worldwide due to its potential health-promoting benefits, such as immune-modulatory, anti-proliferative and anti-inflammatory effects [1], upon certain dietary consumption. However, considering the great nutritional value of honey in combination with its high price and significant market share, it is a commodity highly susceptible to fraudulent practices. Botanical or geographical origin mislabeling, the addition of low-cost syrups and dilution of honey with water are among the most common fraudulent acts [2]. Thus, it is necessary to develop analytical methods to assure honey quality and protect consumers and market sustainability. 

A plethora of analytical techniques has been used to assess honey authenticity. As we comprehensively discussed in our recent publication [3], chromatographic and spectroscopic techniques, alongside conventional methods measuring physicochemical properties, such as electrical conductivity or acidity, are commonly applied in the field. Although spectroscopic methods usually provide non-destructive analysis and conventional methods are widely available due to their low-cost, combining chromatography with mass spectrometry (MS) permits the detection of various analyte classes, e.g., pesticide residues (to check bio-production) [4], sugars (to evaluate quality characteristics towards established regulation, Directive 2001/110/EC) [5] or phenolic compounds (to estimate geographical, botanical or entomological origin) [6,7]. Importantly, phenolic compounds determination in honey authenticity studies can have a binary character, in detail, both as characteristic markers as well as to evaluate the nutritional value of honey due to their bioactivity [8]. Thus, phenolic compound fingerprinting can be used as a tool to investigate both the origin and potential bioactivity of reputable honey types, such as honey produced in Greece. 

Greek honey is generally considered of high quality due to its organoleptic characteristics [9], biological activity [10] and the biodiversity of the Greek countryside, which includes many endemic plant species [11]. In addition, Greece plays a decisive role in EU honey production and exportation. Indicatively, Greece exported 856 tons of honey to third countries outside the EU from January 2020 to August 2020, a 79% increase compared to its total exports during the corresponding period in 2019. Moreover, Greece had the most hives per beekeeper (147 hives per beekeeper, while the EU average is 21), indicating honey production’s impact on the national economy and agriculture (https://ec.europa.eu/info/sites/default/files/food-farming-fisheries/animals_and_animal_products/documents/market-presentation-honey_spring2020_en.pdf, last accessed 11 May 2022). 

Considering the discussed facts, we analyzed a total of 169 honey samples with different botanical origins, namely blossom (*n* = 62) and the unifloral fir (*n* = 10), oak (*n* = 24), pine (*n* = 39) and thyme (*n* = 34) honeys to reveal botanical origin markers of Greek honey through their phenolic compound content. To achieve that, our recently developed ultra-high-performance liquid chromatography hybrid quadrupole time-of-flight mass spectrometry (UHPLC-q-TOF-MS) method [12] was verified, and the analyte target list was updated, including a total of 24 target phenolic compounds. Besides targeted metabolomics, suspect and non-targeted workflows were also utilized. A more comprehensive estimation of the phenolic compound concentration of Greek unifloral honey was accomplished through suspect screening and semi-quantification, while non-target screening provided a diagnostic tool for honey authenticity assessment. All in all, the present study documents the phenolic profile of Greek honey and showcases metabolomic approaches to identify its botanical origin.

## 2. Results and Discussion 

### 2.1. Target Screening

**Figure 1:**
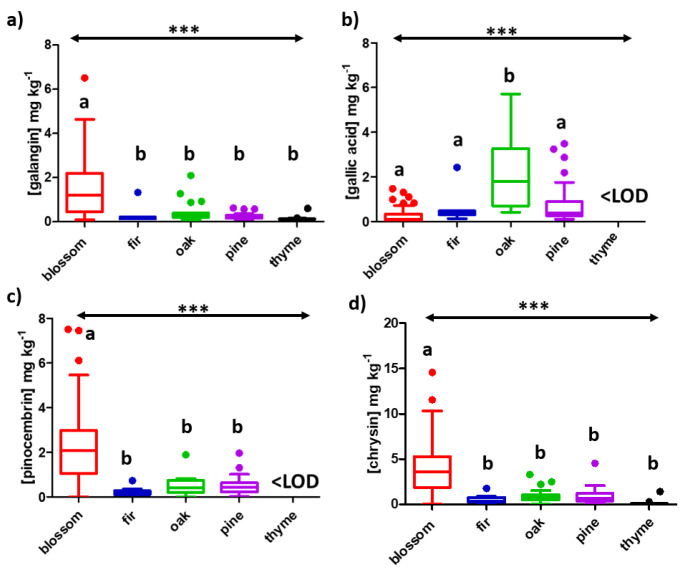
Whisker’s plot for (**a**) galangin, (**b**) gallic acid, (**c**) pinocembrin and (**d**) chrysin performed at the 95% confidence level; ***: *p*-Value < 0.001. Tukey’s multiple comparison test was also performed, and different letters indicate significant differences among the groups.

The developed target list was used to screen and quantify the content of phenolic compounds in all the tested honey samples (Table 1). Whisker’s plots were prepared using the acquired concentrations for all the analytes in every honey matrix to depict the monitored distribution through its quartiles (Figure 1 and Figures S1–S4 in the Supplementary Materials). Importantly, analysis of variance (ANOVA) revealed statistically significant differences (*α* = 0.05) among varieties for the acquired concentrations of all the target analytes, except eriodictyol, luteolin, quercetin, genistein, rosmarinic acid, vanillin and vanillic acid. Actually, 15 out of 17 analytes showed significant differences with a *p*-Value <0.001, whilst *p*-coumaric acid was <0.01 and vanillic acid <0.05 (*α* = 0.05, in all cases). Following ANOVA, a Tukey’s multiple comparison test was performed, revealing four potential botanical origin markers, namely galangin, pinocembrin and chrysin for blossom honey and gallic acid for oak honey (Figure 1). In terms of the blossom honey, a *p*-Value <0.001 was attained in the case of galangin (Figure 1a), pinocembrin (Figure 1c) and chrysin (Figure 1d) against the 4 other botanical origins, while the other groups showed non-significant differences among them. The same pattern was monitored for gallic acid in the case of oak honey (Figure 1b). Galangin, pinocembrin and chrysin have been previously reported in high concentrations in blossom honey (also known as polyfloral honey, blossom honey is a mixture of nectar collected by various plants) and were proposed as discrimination markers of Serbian honey [13]. It is important to mention that these three flavones originate from propolis in the case of European honeys [13]. In addition, all three analytes were identified among the main phenolic compounds in heather blossom honey from Portugal [14] and citrus blossom honey from Spain [15], all Mediterranean countries with similar climatological conditions to Greece. In the case of gallic acid in oak honey, our results are in line with previous studies identifying gallic acid among the main compounds of Turkish oak honey [16]. Actually, oak honey has demonstrated bioactivity inhibiting different enzymes, namely urease and xanthine oxidase [17] as well as hyaluronidase [18]. In fact, in these cases, gallic acid is considered responsible for enzyme inhibition due to its high concentration in oak honey. All in all, it was revealed that by using the developed target list, it was feasible to acquire an indication of which analytes are statistically different per botanical origin, indicating their potential as botanical markers. Of course, to verify this argument, it would be necessary to analyze more samples collected during different years.

### 2.2. Suspect Screening

Through suspect screening, 12 phenolic compounds, namely 2-trans,4-trans-abscisic acid, 2-cis,4-trans-abscisic acid, acacetin, dehydrovomifoliol, homogentisic acid, isokaempferide, isorhamnetin, lumichrome, methyl syringate, phenyllactic acid, sakuranetin and tectochrysin, were identified and semi-quantified in Greek honey varieties. Table S1 summarizes the occurrence of all identified analytes in the analyzed honey varieties.

**Figure 2:**
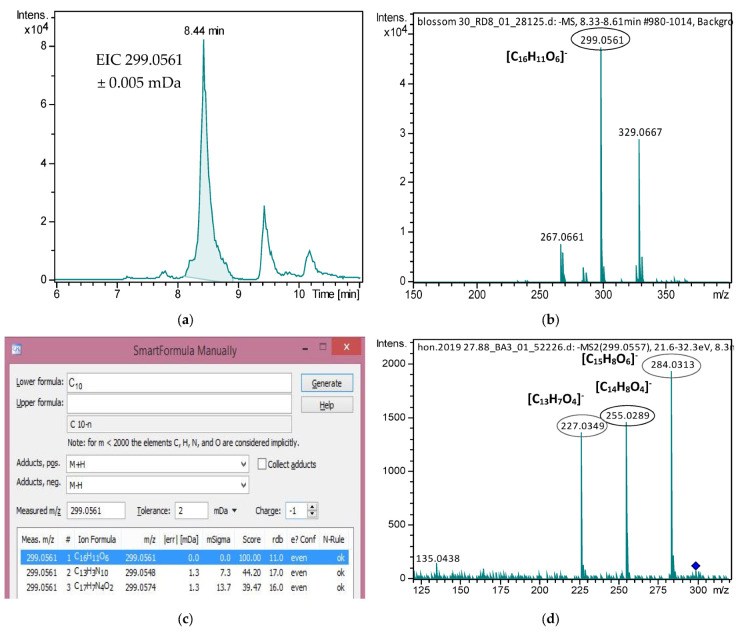

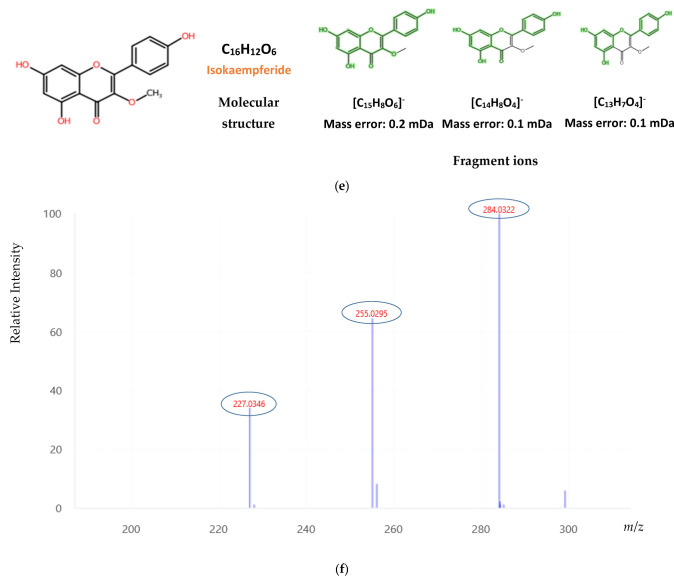
Identification data for the mass feature *m*/*z* 299.0561_8.44 min (Isokaempferide). (**a**) EIC of *m*/*z* 299.0561 in a blossom Honey; (**b**) Background subtracted MS Spectra from 8.3 to 8.6 min; (**c**) Molecular Formula Annotation of *m*/*z* 299.0561; (**d**) DDA MS/MS Spectra of 299.0561; (**e**) Structures of precursor and fragment ions (green part of the structure) of Isokaempferide; (**f**) Vaniya/Fiehn Natural Products Library Record VF-NPL-QTOF008090 (Isokaempferide).

All identified compounds showed high mass accuracy, below 2 mDa, isotoping fitting below 50 mSigma and retention time (tR) tolerance below 0.2 min. Especially for isokaempferide and tectochrysin, which were not included in our previous suspect screening workflow [12], their identification was accomplished by comparing experimental MS/MS fragments with MS/MS spectra found in the mass spectral library MassBank of North America. Additionally, differences between the experimental and predicted tR were considered. The detailed identification data for isokaempferide is presented below (Figure 2), while for tectochrysin, it can be found in Figure S5 of the Supplementary Materials. 

All compounds were semi-quantified using an in-house semi-quantification protocol described in the Section 3. “Materials and Methods”. Specifically, 2-trans,4-trans abscisic acid was semi-quantified with its isomer, 2-cis,4-trans-abscisic acid. For the semi-quantification of homogentisic acid, 3,4-dihydroxybenzoic acid was selected. At the same time, apigenin was the most suitable compound to semi-quantify both isokaempferide and isorhamnetin, while lumichrome and methyl syringate were semi-quantified using eriodictyol. Finally, for phenyllactic acid and tectochrysin semi-quantification, p-coumaric acid and chrysin were selected, respectively. Especially for acacetin, 2-cis,4-trans-abscisic acid and sakuranetin, analytical standards were purchased to verify their identification and reduce uncertainty in determining their concentration. All results are presented in Table 2.

According to the results presented in Table 2, the concentrations of some analytes varied significantly in the different floral varieties. Oak honey was richer in abscisic acid isomers compared to the other varieties. Abscisic acid has been characterized as a main phenolic compound of honeydew honeys [19]. In our study, the greatest amount of 2-cis,4-trans-abscisic acid was found in oak honey, followed by fir honey, with a mean concentration of 1.4 and 0.46 mg/Kg, respectively. These results agree with other studies reporting similar concentration levels for these varieties [20]. Greek thyme honey was richer in methyl syringate than blossom, oak, fir and pine honeys, with methyl syringate being a phenolic compound related to the scavenging activity of superoxides [21]. Methyl syringate was reported in thyme honeys previously [22]. Oak honey was also rich in phenyllactic acid and homogentisic acid, compounds that were previously used for honey authentication [23]. 

All the other suspect compounds were determined in low concentration levels and did not show any potential for discrimination among varieties. Sakuranetin was not identified in pine and fir honeys, while its average content was also low in oak, blossom and thyme honeys. These results are in accordance with previously published studies, with honeydew honeys from New Zealand containing 0.02 mg/Kg and different nectar honeys between 0.0060 and 0.062 mg/Kg [24,25]. Acacetin was not identified in thyme and fir samples; however, it was present in the other three varieties. An average concentration of 0.053 mg/Kg was detected in Greek oak honeys, comparable to Turkish honeys previously studied (0.04 mg/Kg).

Dehydrovomifoliol has also been formerly identified in honey. It has been proposed as a potential marker of heather honey, especially Polish honey [12]. Oak honey was the richest in our results, reaching 1.8 mg/Kg as an average concentration. Another detected suspect compound was isorhamnetin, with an average concentration ranging from 0.033 mg/Kg in thyme samples to 0.11 mg/Kg in oak variety, which was comparable to previous studies [26]. Regarding lumichrome, the highest average concentration was found in blossom honeys; nevertheless, this concentration of 0.33 mg/Kg is significantly lower than the corresponding concentrations reported in Croatian and Italian honey samples [27]. Finally, tectochrysin was identified as a minor constituent in Greek honeys. Tectochrysin has been reported previously in Spanish floral honeys from Galicia with an average concentration of 0.31 mg/Kg, significantly higher than the one calculated in Greek honeys [28]. 

### 2.3. Non-Target Screening

Applying non-targeted screening workflow in honey samples resulted in a bucket table containing 3631 features. Worthwhile to note is that samples collected only during 2016 were processed by the non-targeted screening workflow. Multivariate statistical analysis revealed important features that can be used as authenticity markers to discriminate Greek honey varieties. Score plots, as well as the goodness of fit (R^2^), the goodness of prediction (Q^2^) and model accuracy of each PLS-DA model, can be found in the Supplementary Materials, along with the five most significant variables of each model and their box plots (Figures S6–S26). 

Initially, a PLS-DA model was constructed, attempting to differentiate all 5 honey varieties. Figure S6 shows the final PLS-DA model developed, and the ellipses are drawn at 95% probability level. The score plot shows a distinct separation between blossom and thyme honeys. On the other hand, all samples belonging to the honeydew class (fir, oak and pine) were not well-separated and were distributed between the blossom and thyme groups (Figure S6). This model did not prove to be accurate, although it showed a predictive ability of 0.59 using the first four components. Many factors that increase the variance in the sample set may have influenced model accuracy, such as the different regions from where the samples were collected, the various climatic conditions prevailing in different collecting periods, and the diverse flora occurring in each area [29]. A better separation could have been achieved by using physicochemical parameters and/or melissopalynological analysis as a preliminary step to remove samples that do not comply with specific parameters defined by the legislation (Directive 2001/10/EC). The top five important features with the highest VIP scores were those with monoisotopic masses of 284.0684, 284.1048, 242.1517, 166.0996 and 508.1157 (Figure S7). Their box plots are found in the Supplementary Materials (Figure S8).

Subsequently, identification of the VIPs was undertaken. Indicatively, the following process was applied for the mass feature with *m*/*z* 284.1048 in tR = 9.75 min: First, the molecular formula C_17_H_16_O_4_ was assigned by Smart Formula manually 3D with a 100% score, as it showed a mass error of 0.22 mDa and an isotopic fitting of 27 mSigma. Then, the Compound Crawler tool was used to search public libraries such as ChEBI, Pubchem and ChemSpider. ChEBI library was preferred compared to the others as it incorporates chemical entities of biological interest. However, no candidates were recovered in the ChEBI library, so Pubchem and Chemspider were used, resulting in 17 possible candidates. The candidates were then processed with the in silico fragmentation tool MetFrag using the MS/MS spectra that have been assigned in this feature during data treatment. Metafrag showed the highest score for the candidate phenethyl caffeate, a phenethyl alcohol ester of caffeic acid, which constitutes a bioactive component of honeybee hive propolis, providing many beneficial properties [30]. Its main fragments were explained, namely *m*/*z* 170.0353, 135.0451 and 161.0245, and the molecular features [C_9_H_7_O_4_]^−^ corresponding to the ion formula of caffeic acid, [C_8_H_7_O_2_]^−^, corresponding to the main fragment of caffeic acid (target list, Table S7) and [C_9_H_5_O_3_]^−^ were assigned. As the last step, mass spectral libraries such as MassBank Europe, MassBank of North America and METLIN were searched to find an MS/MS spectrum of a reference standard to reach a higher confidence level in the identification, but it was not available in this case. 

Following the procedure mentioned above, the compound acacetin was assigned in the mass feature of 284.0684 and confirmed by a reference standard. In addition, acacetin was also identified through the suspect screening procedure, and it showed a higher average concentration in blossom honey, in line also with non-target screening findings. Moreover, the mass feature with *m*/*z* 508.1157 corresponds to the [2M − H]^−^ ion of chrysin, an important compound already detected and quantified through the targeted screening. Finally, the mass features with *m*/*z* 242.1517 and 166.0996, which show a higher intensity in thyme honey, are in-source fragments of the *m*/*z* 301.1656, as proposed by metaboscape (Figure S27). The molecular formula C_15_H_26_O_6_ was assigned to the mass feature *m*/*z* 301.1656. A total of 2232 candidates were found in PubChem based on the given molecular formula, and further information (such as intense and clean MS/MS fragments) was needed to proceed to higher identification confidence. Therefore, this mass is tentatively identified at identification confidence level 4, according to the categorization proposed previously [12]. All annotations of important features of all PLS-DA models are presented in Table 3.

A new model was created after removing the samples belonging to the honeydew class in order to investigate the discrimination between blossom and thyme varieties. Thyme, one of the most widespread and important Greek unifloral honeys, is usually degraded by adding, without declaration, other nectar honeys or blossom honey and mislabeled as thyme honey [31]. Thus, it is of paramount importance to create a model to certify its authenticity. A highly accurate model leading to R^2^ = 0.99587 and Q^2^ = 0.81258 was achieved using the first five components (Figure S9). The first five variables in descending order were 508.1157, 544.1364, 284.0684, 286.0841 and 314.0790, which shows a higher concentration level in blossom honeys (Figure S10). Their box plots can be found in the Supplementary Materials (Figure S11). The monoisotopic masses 508.1157 and 284.0684 were common with the previous model, and they correspond to [2M − H]^−^ of chrysin and acacetin, respectively. The mass features with *m*/*z* 544.1364 and 314.0790 were annotated as [2M − H]^−^ of pinobanksin and pinobanksin 3-O-acetate, respectively. Both of the MS/MS spectra of these features contain the fragments with *m*/*z* 271.0612, 253.0495, 197.0597 and 225.0546, corresponding to the pseudomolecular ion of pinobanksin and its main fragments (Table S7). Pinobanksin constitutes a significant phenolic compound found in different honey matrices and provides many beneficial properties to human health [12]. As revealed in the targeted screening results, pinobanksin showed a much higher average concentration in blossom honeys and can be considered a potential biomarker for this variety. Finally, for the mass feature with *m*/*z* 286.0841, the molecular formula C_16_H_14_O_5_ was assigned; however, this led to many possible candidates, making its unequivocal identification very difficult. 

Since it was not feasible to attain sample discrimination using all five different honey classes, in the next step, we worked with binary discriminatory models to reveal significant compounds found in these varieties. Thus, two classes were formed in each model. The first one included the samples from a selected variety, while the second contained the rest of the samples. So, five models were developed. PLS-DA score plots, cross-validation details, important features, and their box plots are presented in the Supplementary Materials (Figure S12–S26). Τhe most satisfactory results in terms of accuracy and predictive ability were obtained in the separation models of thyme and blossom categories with Q^2^ = 0.82468 and Q^2^ = 74198, respectively. On the other hand, fir, pine and oak models showed lower predictive ability ranging from 0.58 to 0.64. Honeydew honeys are often more difficult to differentiate as their composition depends mainly on the beekeeping plants that exist in the forests of each region. Therefore, honeydew honey may contain a high portion of nectar, and its composition must be checked so that it complies with the EU legislation (Directive 2001/10/EC). 

In the discrimination model of blossom honeys, two important markers were identified by comparing their MS/MS spectra with those obtained by in silico fragmentation. The first marker was phenethyl caffeate, a compound that has already been mentioned before as a significant feature of Greek blossom honey [30]. The second marker, prenyl caffeate, is also a caffeic acid derivative, and it has been reported in Serbian polyfloral [13] samples and Algerian honeys [29]. Other markers could not be identified as many possible structures were retrieved for their assigned molecular formula. Regarding the thyme discrimination model, eudesmic acid was recognized by comparing experimental MS/MS data with those in the MoNA spectral library, while 3-methylgalangin with in silico MS/MS data produced by Metfrag. Eudesmic acid is cited as an important compound for Manuka honey, while 8-methylgalangin has been reported for Chilean honeys [32,33]. The oak model revealed three important markers. As previously mentioned, gallic acid is a marker for oak honey samples and was identified with a reference standard. Scopoletin has formerly been reported in cotton honey [34], and its identity was confirmed by comparison with the MoNA library. Finally, taxifolin proved to be an important compound for Greek pine honeys, confirmed by target screening results. 

## 3. Materials and Methods

### 3.1. Chemicals

All chemicals, solvents and reagents used were of high analytical purity. Ammonium acetate, sodium sulfate anhydrous and EtAc (purity 99.0% or greater) were purchased from Sigma-Aldrich, while ACN and LC-MS grade methanol (MeOH) were provided by Merck. A Milli-Q Millipore system purification system (Direct-Q UV, Millipore, Bedford, MA, USA) was used to prepare the aqueous solutions. Additionally, 2,5 dihydroxybenzoic acid (purity ≥99.0%), 3,4 dihydroxybenzoic acid (purity ≥97.0%), 4 hydroxybenzoic acid (purity ≥99%), apigenin (purity ≥99%), caffeic acid (purity ≥98.0%), chrysin (purity ≥99.0%), cinnamic acid (purity ≥99%), eriodictyol (purity ≥98.0%), ferulic acid (purity ≥99%), galangin (purity ≥99.0%), gallic acid (purity ≥99.0%), genistein (purity ≥99.0%), luteolin (purity ≥97.0%), naringenin (purity ≥99.0%), p-coumaric acid (purity ≥98.0%), pinobanksin (purity ≥99.0%), pinocembrin (purity ≥99.0%), quercetin (purity ≥95%), rosmarinic acid (purity ≥99.0%), salicylic acid (purity ≥99.0%), syringic acid (purity ≥98%), taxifolin (purity ≥85%), vanillic acid (purity ≥97%), vanillin (purity ≥99%), acacetin (purity ≥97%), sakuranetin (purity ≥98%) and 2-cis,4-trans-abscisic acid (purity ≥98%) were purchased by Sigma-Aldrich (St. Louis, MO, USA). Finally, regenerated cellulose syringe filters (R.C. filters, pore size 0.2 μm, diameter 15mm) were acquired from Phenomenex (Torrance, CA, USA).

### 3.2. Standard Preparation

A 1000 mg L^−1^ in MeOH stock solution was prepared for each target compound. Then, the standards were stored at −20 °C in amber glass bottles to avoid photodegradation. Two mixture working solutions at two different concentrations, 25 and 50 mg L^−1^, were also prepared and stored in the refrigerator. Dilution of the stock solution with mobile phase, MeOH: H_2_O (50:50), yielded working solutions at 0.25, 0.50, 1.0, 2.0, and 5.0 mg L^−1^. Calibration curves were obtained by plotting the peak areas of the standards against their concentration. Similar calibration curves were constructed in a blank honey matrix (matrix-matched calibration curve) to assess important method performance characteristics, namely, linearity, precision and matrix effects as well as analyte quantification. The matrix-matched standards were prepared by spiking the compounds in a blank honey extract prior to injection.

### 3.3. Honey Samples and Sample Preparation

One hundred and sixty-nine (*n* = 169) Greek honey samples from blossom (*n* = 62), fir (*n* = 10), oak (*n* = 24), pine (*n* = 39) and thyme (*n* = 34) varieties were collected by ATTIKI Honey SA from different regions of Greece (see supplementary material for further details, Table S2). Importantly, the samples were collected and analyzed during two consecutive years (2016 and 2017) to enhance the variability of the tested parameters. The samples were stored in amber glass containers at 4 °C. Before analysis, the samples were mixed vigorously for 3 minutes to be homogenized and in case of crystallization, samples were put in a water bath at 40 °C till they were liquified. Sample preparation was based on a previously published study of our group [12]. Briefly, 1 g of honey sample was weighted in a 15 mL centrifuge tube and diluted with 5 mL of acidified water (pH < 2) containing 2% sodium chloride. After vortexing for 1 min, the samples were extracted three times with 5 mL EtAc. The samples were centrifuged between each extraction step to better separate the two phases. The combined extracts were collected in a glass tube and dried with anhydrous sodium sulfate. Afterwards, the extracts were evaporated under a gentle nitrogen stream to dryness and then reconstituted to 0.2 mL with a final proportion of MeOH:H_2_O (50:50). Before being injected into the HPLC system, all samples were filtered using cellulose syringe filters (R.C. filters, pore size 0.2 μm, diameter 15mm). In addition, to assess potential drifts and evaluate the reproduction of the analysis, a quality control (QC) sample was prepared by mixing 20 μL of each honey sample extract to attain analytical information from all different botanical and geographical origins. Finally, ultrapure water was used to prepare a procedural blank, subjecting the whole sample preparation protocol to subtract possible contamination during data processing.

### 3.4. Method Verification

To monitor the analytical performance of the method, all the necessary quality performance characteristics were investigated, namely trueness, repeatability, intermediate precision, selectivity, linearity, limits of detection (LODs) and limit of quantification (LOQs). Considering that the current study is based on an in-house validated method [12], on this occasion, method verification was performed and seven additional analytes were included in the target list, namely, chrysin, galangin, genistein, naringenin, pinobanksin, pinocembrin and rosmarinic acid, resulting in a target list of 24 analytes. All the investigated performance characteristics were calculated as described in our previous paper [12], and detailed verification results can be found in the (Supplementary Material Tables S3–S5).

### 3.5. UPLC-QToF-MS Analysis

A UHPLC system (Dionex UltiMate 3000 RSLC, Thermo Fisher Scientific) coupled with a Q-ToF MS (Maxis Impact, Bruker Daltonics) was utilized. The chromatographic separation was performed on an Acclaim RSLC C18 column (2.1 × 100 mm, 2.2 μm) from Thermo Fischer Scientific (Waltham, MA, USA), equipped with an Acquity UPLC BEH C18 VanGuard Pre-Column from Waters (Milford, MA, USA) at 30 °C. The mobile phase mixtures comprised (A) Milli-Q H_2_O: MeOH (90:10) and (B) MeOH, both A and B containing 5 mM ammonium acetate. The LC gradient elution and flow rate program is described in Table S6 of the Supplementary Materials. The injection volume was set to 5 µL. The QToF-MS system was equipped with an ESI source, operating in negative ionization mode. The operation parameters for ESI were set as follows: capillary voltage, 3500 V; endplate offset, 500 V; nebulizer gas pressure 2 bar (N2); drying gas, 8 L min^−1^ and dry temperature, 200 °C. 

The QTOF-MS system was operating in broadband collision-induced dissociation (bbCID) acquisition mode and recorded spectra over the *m*/*z* range 50–1000 with a scan rate of 2 Hz. The Bruker bbCID mode is a data-independent acquisition mode (DIA) that provides MS and MS/MS spectra at the same time, working at two different collision energies; at low collision energy (4 eV), MS spectra are acquired, while at high collision energy (25 eV), MS/MS spectra are collected. In addition, honey samples from each botanical and geographical origin, as well as the pool QC sample, were also analyzed using a data-dependent acquisition mode (DDA), AutoMS. In AutoMS, the five most abundant ions per MS scan are selected and fragmented, providing precise and compound-specific MS/MS spectra. Thus, this mode is most suitable for the structure elucidation of unknowns.

A QTOF-MS external calibration was performed before analysis with a 10 mM sodium formate solution in a mixture of water/isopropanol (50:50). The exact theoretical masses of calibration ions with formulas HCOO(NaCOOH)1-14 in the range of 50–1000 Da were used for calibration. Internal calibration was also performed using a calibrant injection at the beginning of each run in a dedicated calibration segment (0.1–0.25 min).

### 3.6. Targeted, Suspect and Non-Targeted Screening Workflows 

An accurate-mass target screening database was compiled and used to identify and quantify 24 phenolic compounds in all honey samples (see Supplementary Materials, Table S7). Briefly, the identification criteria were as follows: retention time tolerance lower than ±0.2 min, mass accuracy of the precursor and qualifier ions less than 5 mDa, isotopic fit less or equal than 50 mSigma (Bruker mSigma is a measure of the goodness of fit between the measured and the theoretical isotopic pattern) and the existence of at least two qualifier ions. The target screening was performed using the software TASQ 1.4 and DataAnalysis 4.4 (Bruker Daltonics, Bremen, Germany) along with other tools included in this software, such as Bruker Compass Isotope Pattern and SmartFormula Manually. Instead of external standard calibration curves for analyte quantification, matrix-matched calibration curves were used to compensate for ion suppression or enhancement in the ionization source. To identify statistically significant differences among the five botanical groups based on the attained concentration of each target analyte, ANOVA followed by Tukey’s multiple comparison test was performed at a significance level, *α* = 0.05 using GraphPad prism 5.0 software (San Diego, CA, USA). Additionally, to visualize the variance of the attained concentration for each target analyte, Whisker’s plots are provided, developed in GraphPad prism 5.0 software.

The suspect screening workflow is thoroughly described in a previously published work [12]. Firstly, eight compounds contained in the suspect list from our previous work were bought and incorporated into the target list. Then, the suspect list was enlarged by adding eight more compounds, namely, isokaempferide, caffeic acid isoprenyl ester, (−)-epigallocatechin gallate, 4-hydroxyphenylacetic acid, arbutin, baicalein, astragalin and kynurenic acid that have been mentioned to exist in honey. Information about formulas, monoisotopic masses and pseudomolecular ions, possible fragment and adduct ions as well as the predicted retention times [35] for these specific compounds are meticulously presented in Supplementary Materials (Table S8, [12,22,36,37,38,39,40,41]). Furthermore, the experimental retention times for the compounds that have been previously identified have also been added to the suspect list and used to increase identification confidence. Then, the final list, containing 60 compounds, was used to screen the honey samples to gain a deeper knowledge of phenolic compounds in Greek varieties. Mass accuracy threshold of 5 mDa, isotopic fit below or equal to 50 mSigma, ion intensity more than 1000 and peak area threshold of more than 2000 were utilized for creating the extracted ion chromatogram. For this purpose, the program TASQ 1.4 (Bruker Daltonics, Bremen, Germany) was used.

The identification workflow was followed for all compounds found in the suspect list. Briefly, MS spectra were meticulously examined for the existence of possible in-source fragments and/or adducts, and a formula for the precursor ion was proposed from Smart Formula manually. Then, MS/MS spectra were compared to those that exist in mass spectral libraries such as Fiehn Lab MassBank of North America (https://mona.fiehnlab.ucdavis.edu/, last accessed 8 June 2022) and METLIN [42] or by in silico fragmentation tools such as MetFrag [43]. Furthermore, experimental retention times were compared with theoretical to distinguish potential isomers and eliminate false positive results [12].

The identified suspect compounds were then semi-quantified in order to estimate their concentration levels in Greek honey samples. A popular way to perform semi-quantification is to use similar structural compounds found in the target list; however, this is not the most suitable method as important parameters such as logD and RT are not considered [44]. A more accurate way is to search for structurally similar compounds based on the number of similar functional groups, as well as the distance between functional groups. The online tool, ChemMine (https://chemminetools.ucr.edu/, last accessed 8 June 2022) was used for this goal. The Smile of each suspect compound was imported, and the comparison with compounds found in the PubChem database ensued, using a similarity cutoff of 0.9. The analytes with the highest structural similarity were recorded and the target compound with the higher similarity score was selected for semi-quantification. The similarity score is calculated as the Tanimoto similarity of substructure-based fingerprints [44].

Non-target screening workflow was performed using Bruker Metaboscape 3.0, an integrated software capable of implementing the entire procedure from peak peaking to multivariate analysis. The workflow contains automatic calibration using a calibrant (sodium formate solution) injection at the start of each run, as well as non-linear retention time alignment using T-ReX 3D, which connects isotopes, adducts and fragments of each feature together. The parameters used for bucket table formation were as follows: intensity threshold higher than 2000 counts, minimum peak length of 8 spectra, a mass range of 50–1000 *m*/*z*, Rt range of 0.5–12 min, an extracted ion chromatograms (EIC) correlation of 0.8 (only features above this threshold can be treated as adducts or fragments), primary ion [M − H]^−^, seed ion [M + Cl]^−^ and common Ion [M − H − H_2_O]^−^. Background features were discarded by subtracting procedural blank runs. Furthermore, if a feature is not found in at least 25% of the samples of a group is also removed [45]. 

Multivariate statistical analysis was performed to spot new markers that can differentiate the samples according to their botanical origin. The bucket table produced by Metaboscape, after blank buckets removal, was exported as a csv file. Then, multivariate statistical analysis was performed by importing this file into the online platform Metaboanalyst 5.0, a dedicated platform for metabolomics applications. Firstly, the group labels were added to all samples, and missing values were replaced by 1/5 of min positive values of their corresponding variables. Data filtering was not used, and the bucket table was normalized by sum to reduce systematic variation among samples. In addition, data were transformed using the logarithm function and scaled using the auto-scaling algorithm, which mean-centered the data and divided the standard deviation of each variable. Finally, Partial Least Squares Discriminant Analysis (PLS-DA) was used to discriminate the different varieties and obtain the most influential variables that can be used as markers. Thus, the features with the 5 higher VIP scores of the PLS-DA model were kept and further examined to be identified. Ten-fold cross-validation (CV) was performed where the whole dataset was divided into 10 parts, 9 parts were used for training the model, and 1 part was kept as a test set. This procedure happens 10 times, and the total error is the mean of the errors produced after each test. As a model performance measure, the Q^2^ was used, which is the model’s predictive ability.

Complementary to the previous model, 5 PLS-DA two-class models were created, each one referring to the discrimination of a class compared to the rest of the samples. These models were built to find important variety-specific biomarkers that discriminate each variety. The workflow was similar to that described above and included one more step. Before obtaining the csv file used for building each model and importing it in Metaboanalyst 5.0, a t-test significance test was implemented in Metaboscape 3.0 to keep only statistically significant variables with *p* < 0.05. Thus, a file containing the intensities of all samples for the selected buckets was acquired, and the multivariate statistical analysis ensued.

The identification of the above-mentioned important features was based on the interpretation of MS and MS/MS data, with Metaboscape software providing all the necessary tools. Firstly, the target list containing information about standard compounds was imported into Metaboscape as an analyte list, helping to identify some buckets based on specific criteria such as mass accuracy, isotopic fitting, tR tolerance and existence of fragments ions. Another vital tool was Smart Formula manually 3D, with which molecular Formulas were assigned to possible markers in terms of mass accuracy and isotoping fitting. After molecular formula assignment, potential compounds were retrieved using Compound Crawler by public libraries such as PubChem and ChemSpider. Finally, a comparison between experimental MS/MS data with in silico fragmentation produced by Metrfag ensued. Spectral libraries such as Mass Bank Europe, Massbank of North America and Metlin were used to reach a higher level of confidence in the identification. 

## 4. Conclusions

The application of the various HRMS metabolomic approaches provided successful discrimination of Greek honey from five different botanical sources. Targeted, suspect and non-targeted workflows were utilized, providing fruitful information on the Greek honey polyphenolic compound profile. In terms of targeted analysis, it was proven that potential botanical origin markers could be attained even by using univariate analysis, i.e., ANOVA. Nevertheless, analytical standards are used to quantify the targeted analytes, increasing the method cost. Suspect screening can be used complementary to targeted analysis to acquire a detailed characterization of the honey metabolomic profile. Semi-quantification is also possible by using a few analytical standards based on the structural similarity of the analytes. In contrast to the previous two cases, the non-targeted analysis did not require any analytical standards, and further enhanced sample clustering based on advanced chemometrics. Overall, the present study provides a wealth of knowledge on the metabolomic composition of Greek honey, and the proposed markers may be used to make national honey production more secure. 

## Figures and Tables

**Table 1:** Targeted screening results expressed as median concentration (mg kg^−1^) of phenolic compounds in the 5 different honey matrices.

Compound	LOD	Blossom, *n* = 62	Fir, *n* = 10	Oak, *n* = 24	Pine, *n* = 39	Thyme, *n* = 34
2,5 dihydroxybenzoic acid	0.070	0.21	0.71	0.82	0.92	0.11
3,4 dihydroxybenzoic acid	0.083	1.0	3.4	10	4.7	0.50
4 hydroxybenzoic acid	0.098	1.5	1.3	1.0	1.8	0.35
apigenin	0.082	0.15	0.10	0.12	0.11	0.17
caffeic acid	0.065	0.77	0.21	0.38	0.49	0.080
chrysin	0.032	3.6	0.35	0.72	0.65	0.061
cinnamic acid	0.043	0.26	0.17	0.24	0.13	0.022
eriodictyol	0.048	0.098	<LOD	0.10	0.28	0.10
ferulic acid	0.030	0.58	0.21	0.27	0.37	0.045
galangin	0.070	1.2	0.15	0.27	0.21	0.12
gallic acid	0.067	0.092	0.37	1.8	0.37	<LOD
genistein	0.081	0.089	<LOD	0.098	0.10	0.11
luteolin	0.079	0.14	0.34	0.15	0.16	0.14
naringenin	0.050	1.4	0.25	0.59	0.60	0.073
p-coumaric acid	0.16	0.60	0.59	0.72	0.94	0.27
pinobanksin	0.055	1.4	0.25	0.57	0.59	0.069
pinocembrin	0.076	2.1	0.15	0.40	0.44	<LOD
quercetin	0.067	0.15	0.49	0.20	0.18	0.15
rosmarinic acid	0.084	<LOD	0.14	<LOD	<LOD	0.12
salicylic acid	0.33	0.76	0.81	1.6	1.3	0.34
syringic acid	0.081	<LOD	<LOD	0.25	0.36	<LOD
taxifolin	0.084	0.18	0.32	0.20	0.41	0.23
vanillic acid	0.12	0.12	0.15	<LOD	0.12	<LOD
vanillin	0.037	0.50	0.16	0.27	0.59	0.098

LOD: limit of detection.

**Table 2:** Semi-quantification of the analytes identified through suspect screening in the 5 different honey matrices.

Analytes	Honey Matrix									
	Blossom		Fir		Oak		Pine		Thyme	
	Mean (mg/Kg)	SD	Mean (mg/Kg)	SD	Mean (mg/Kg)	SD	Mean (mg/Kg)	SD	Mean (mg/Kg)	SD
Acacetin	0.092	0.13	ND	-	0.053	0.032	0.062	0.043	ND	-
2-trans,4-trans-abscisic acid	0.21	0.37	0.31	0.58	0.92	1.4	0.17	0.19	0.12	0.052
2-cis,4-trans-abscisic acid	0.37	0.58	0.46	0.88	1.4	1.8	0.33	0.40	0.19	0.12
Sakuranetin	0.024	0.030	0.010	0.010	0.022	0.011	ND	-	ND	-
Homogentisic acid	0.41	1.0	0.051	0.12	0.93	2.1	0.41	1.7	0.30	0.79
Dehydrovomifoliol	1.1	2.2	0.72	1.1	1.8	2.2	1.7	1.9	1.04	1.2
Isokaempferide	0.040	0.061	ND	-	0.023	0.021	0.023	0.030	ND	-
Isorhamnetin	0.071	0.12	0.10	0.13	0.11	0.084	0.074	0.14	0.033	0.043
Lumichrome	0.33	1.1	ND	-	0.11	0.15	0.29	0.55	0.15	0.36
Methyl Syringate	0.78	1.5	0.33	0.53	0.65	1.1	0.32	0.49	1.4	1.9
Phenyllactic acid	1.8	2.7	0.96	1.0	4.7	4.1	2.8	3.0	1.4	1.2
Tectochrysin	0.049	0.091	ND	-	0.030	0.030	0.024	0.043	ND	-

SD: standard deviation, ND: not detected.

**Table 3:** Identification of Important markers proposed by PLS-DA models.

PLS-DA Model	Variable (*m*/*z*, Monoisotopic Mass)	VIP Value	Rt (min)	Molecular Formula	Δ*m*/*z* [mDa]	mSigma	Name
All varieties	284.0685	4.52	10.14	C_16_H_12_O_5_	0.159	5.6	Acacetin
	284.1048	4.29	9.75	C_17_H_16_O_4_	−0.222	6.9	Phenethyl caffeate
	242.1517	4.21	4.72	C_13_H_22_O_4_	−0.437	21.0	Unknown 1
	166.0996	4.15	4.72	C_10_H_14_O_2_	0.021	3.6	Unknown 2
	508.1157	4.13	9.68	C_30_H_20_O_8_	−0.567	35.8	[2M − H]^−^ of Chrysin
Blossom vs. Thyme	508.1157	5.50	9.68	C_30_H_20_O_8_	−0.567	35.8	[2M − H]^−^ of Chrysin
	544.1364	5.35	7.25	C_30_H_24_O_10_	1.912	38.8	[2M − H]^−^ of Pinobanksin
	284.0685	5.06	10.14	C_16_H_12_O_5_	0.159	5.6	Acacetin
	286.0841	4.65	6.97	C_16_H_14_O_5_	−0.125	11.2	uknown 1
	314.0790	4.57	9.01	C_17_H_14_O_6_	1.010	4.6	Pinobanksin 3-O-acetate
Blossom	237.0999	3.34	4.07	C_9_H_17_O_7_	0.495	32.3	Unknown 1
	284.1048	3.20	9.75	C_17_H_16_O_4_	−0.222	6.9	Phenethyl caffeate
	248.1047	2.57	9.45	C_14_H_16_O_4_	−0.257	15.2	Prenyl caffeate
	364.1522	2.56	4.89	C_19_H_24_O_7_	0.612	17.9	Unknown 2
	378.1673	2.49	5.05	C_20_H_26_O_7_	−0.032	36.6	Unknown 3
Thyme	212.0686	3.50	3.55	C_10_H_12_O_5_	0.215	7.8	Eudesmic acid
	282.1094	3.43	5.59	C_14_H_18_O_6_	0.845	10.5	Unknown 1
	242.0579	3.21	5.72	C_14_H_10_O_4_	−0.217	6.1	Unknown 2
	284.0684	3.05	9.92	C_13_H_22_O_4_	0.074	7.7	3-methylgalangin
	242.1517	3.04	4.72	C_16_H_12_O_5_	−0.437	12.9	Unknown 3
Fir	130.0631	4.03	1.34	C_6_H_10_O_3_	−1.607	34.2	unknown 1
	124.0164	3.28	1.68	C_6_H_4_O_3_	0.961	11.2	unknown 2
	160.1090	2.92	5.61	C_8_H_16_O_3_	−0.755	14.9	unknown 3
	62.9962	2.88	4.09	-	-	-	unknown 4
	120.0416	2.87	2.55	C_4_H_8_O_4_	−1.835	32.2	unknown 5
Oak	222.0891	4.73	4.34	C_12_H_14_O_4_	1.808	13.7	unknown 1
	192.0424	4.47	4.95	C_10_H_8_O_4_	1.486	7.7	scopoletin
	278.1278	4.43	4.43	C_19_H_18_O_2_	1.619	17.8	unknown 2
	170.0215	4.26	1.27	C_7_H_6_O_5_	−0.002	12.7	gallic acid
	292.0211	4.25	3.47	C_20_H_4_O_3_	−1.151	31.0	unknown 3
Pine	378.1673	2.74	5.05	C_20_H_26_O_7_	−0.032	36.6	unknown 1
	516.1989	2.63	4.20	C_27_H_32_O_10_	1.281	46.0	unknown 2
	264.0786	2.53	7.08	C_17_H_12_O_3_	0.066	4.8	Unknown 3
	350.1364	2.43	4.25	C_18_H_22_O_7_	−0.275	4.8	Unknown 4
	304.0582	2.39	4.88	C_15_H_12_O_7_	−0.037	23.6	Taxifolin

## Data Availability

Data are available upon request.

## Supplementary Materials

Detailed experimental results information can be downloaded at: https://www.mdpi.com/article/10.3390/molecules27144444/s1.

## Sample Availability

Not available.
