# Supplementary material for: Thorough Investigation of the Phenolic Profile of Reputable Greek Honey Varieties: Varietal Discrimination and Floral Markers Identification Using Liquid Chromatography–High-Resolution Mass Spectrometry
Source: Molecules. 2022 Jul 11;27(14):4444. doi: 10.3390/molecules27144444 (PMC9323402; doi:10.3390/molecules27144444)
Supplement: Supplementary file 1 [file molecules-27-04444-s001.zip › molecules-1786070-supplementary.pdf]

ELECTRONIC SUPPLEMENTARY MATERIAL

Thorough investigation of phenolic profile of reputable Greek honey varieties: Varietal discrimination and floral markers identification using Liquid Chromatography - High Resolution Mass Spectrometry

Georgios A. Koulis, Aristeidis S. Tsagkaris, Panagiota A. Katsianou, Panagiotis-Loukas P. Gialouris, Ioannis Martakos, Fotis Stergiou, Alberto Fiore, Eleni I. Panagopoulou, Sofia Karabournioti, Carsten Baessmann, Noud van der Borg, Marilena Dasenaki, Charalampos Proestos and Nikolaos S. Thomaidis

1. Whisker's plots for the target analytes

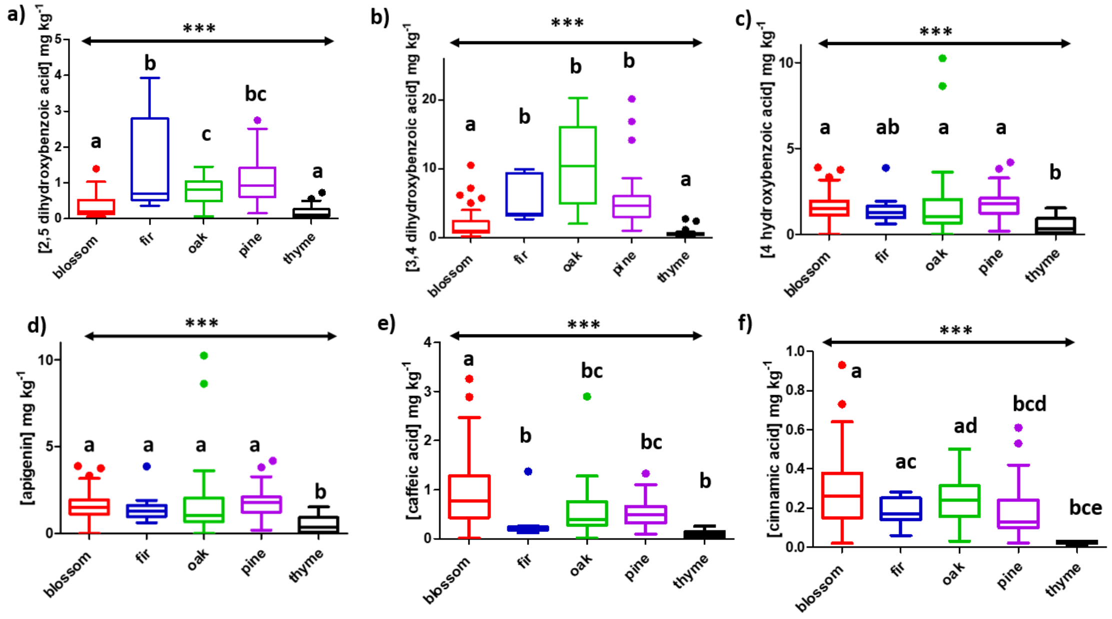

Figure S1. Whisker's plot for a) 2,5-dihydroxybenzoic acid, b) 3,4-dihydroxybenzoic acid, c) 4-hydroxybenzoic acid, d) apigenin, e) caffeic acid and f) cinnamic acid performed at the 95% confidence level; ***: p-value< 0.001. Tukey's multiple comparison test was also performed and different letters indicate significant differences among the groups.

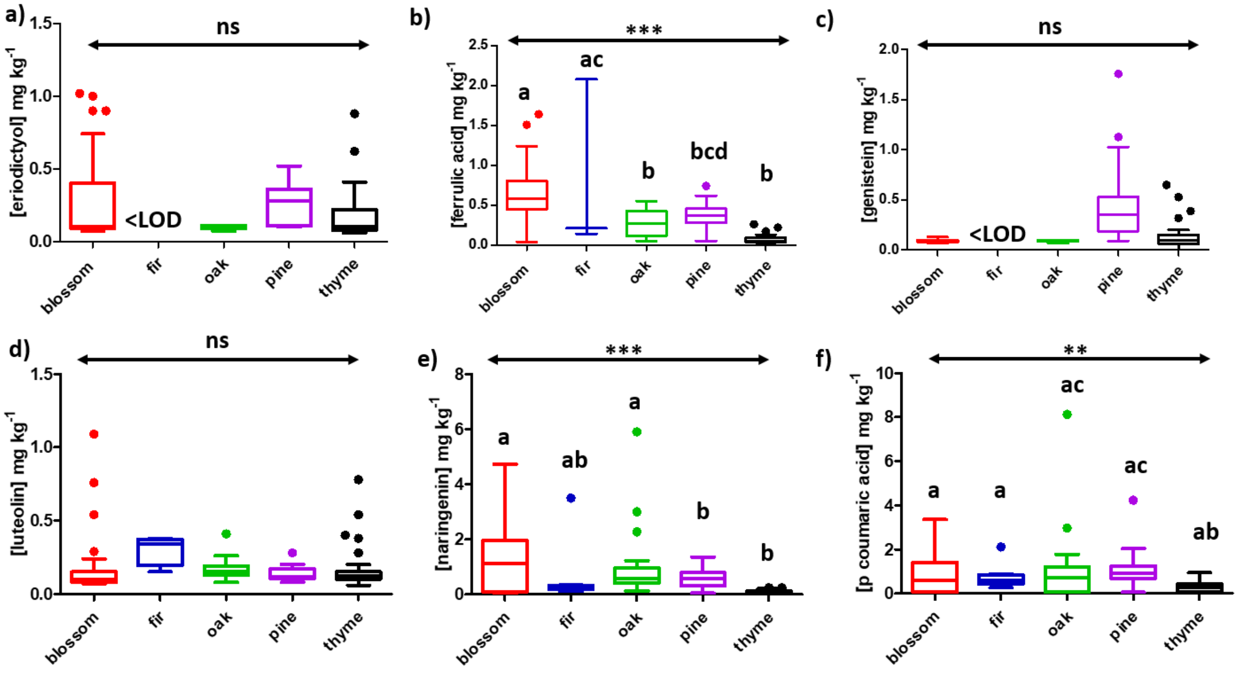

Figure S2. Whisker's plot for a) eriodictyol, b) ferulic acid c) genistein, d) luteolin, e) naringenin and f) p-coumaric acid performed at the 95% confidence level; ***: p-value< 0.001, **: p-

value<0.01. Tukey's multiple comparison test was also performed and different letters indicate significant differences among the groups.

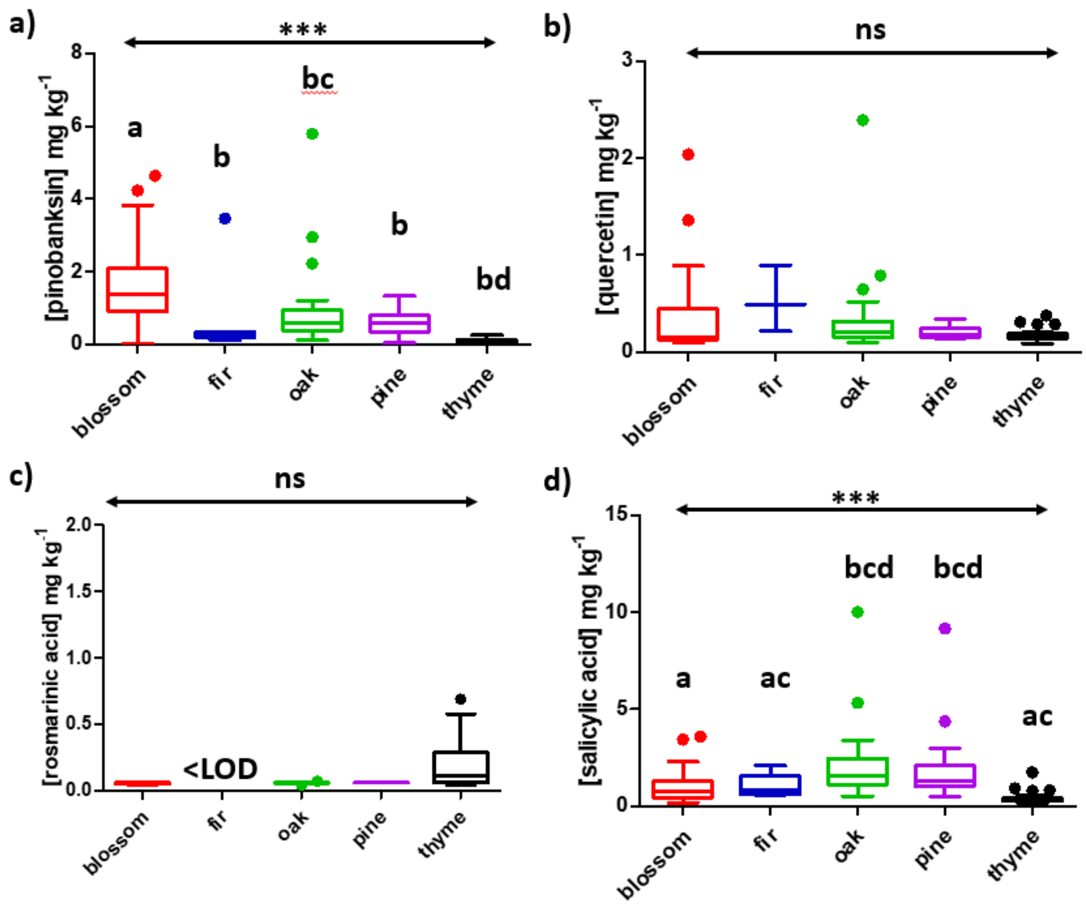

Figure S3. Whisker's plot for a) pinobanksin, b) quercetin, c) rosmarinic acid and d) salicylic acid performed at the 95% confidence level; ***: p-value< 0.001. Tukey's multiple comparison test was also performed and different letters indicate significant differences among the groups.

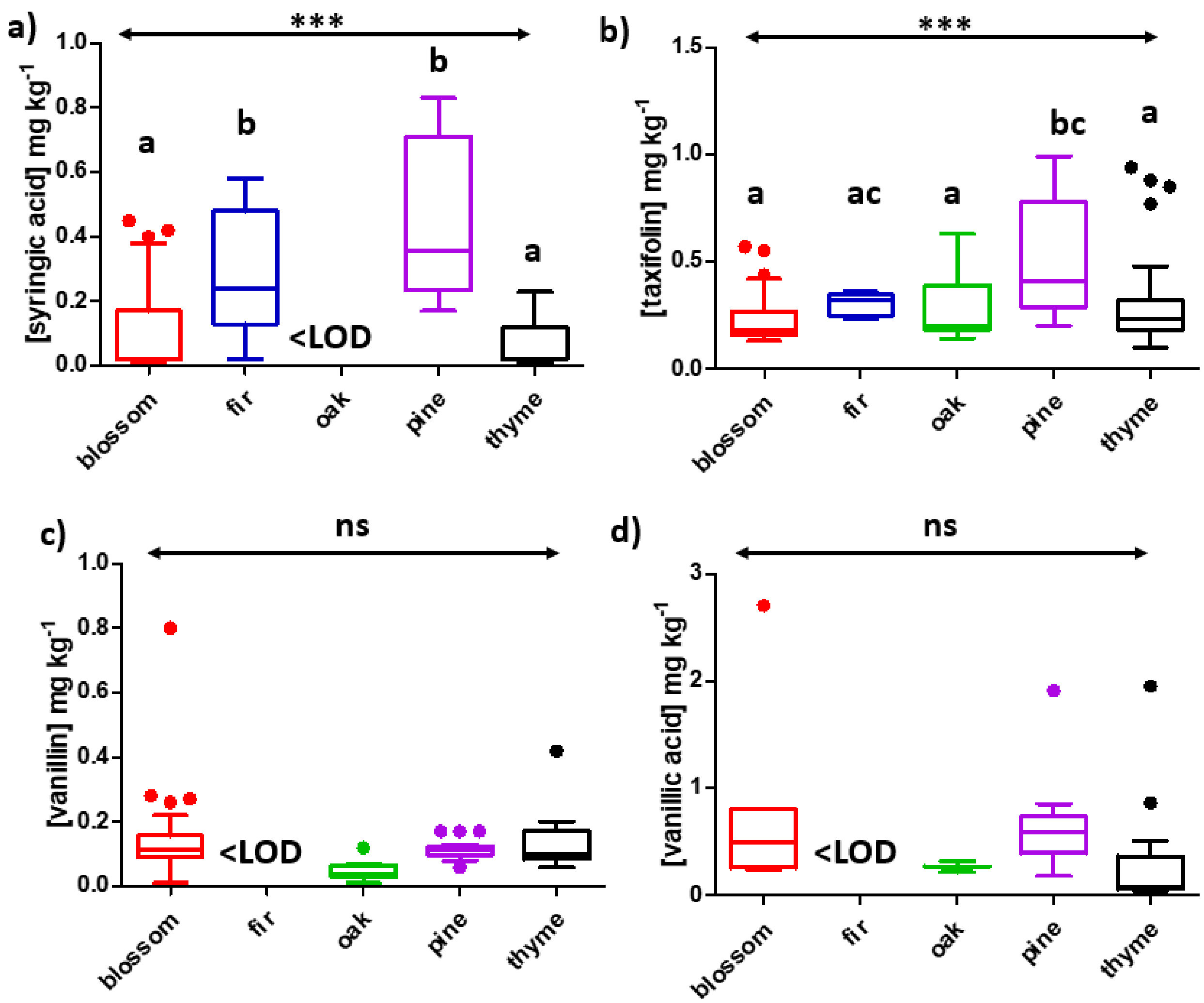

Figure S4. Whisker's plot for a) syringic acid, b) taxifolin c) vanillin and d) vanillic acid performed at the 95% confidence level; ***: p-value< 0.001. Tukey's multiple comparison test was also performed and different letters indicate significant differences among the groups.

Supporting information for suspect screening results

Table S1. Number of samples of each botanical origin that have been identified each compound

Compound	Blossom (#62)	Oak (#24)	Pine (#39)	thyme (#34)	fir (#10)
2-cis,4-trans-abscisic acid	39	24	31	25	9
2-trans,4-trans-abscisic acid	29	22	24	12	8
Acacetin	54	24	36	1	9
Dehydrovomifoliol	49	22	36	30	9
Homogentisic Acid	9	6	5	5	1
Isokaempferide	41	15	14	0	0
Isorhamnetin	34	20	18	15	7
Lumichrome	17	10	18	10	0
Methyl Syringate	28	11	17	26	4
Phenyllactic Acid	41	24	35	30	9
Sakuranetin	45	19	24	3	3
Tectochrysin	37	10	13	0	1

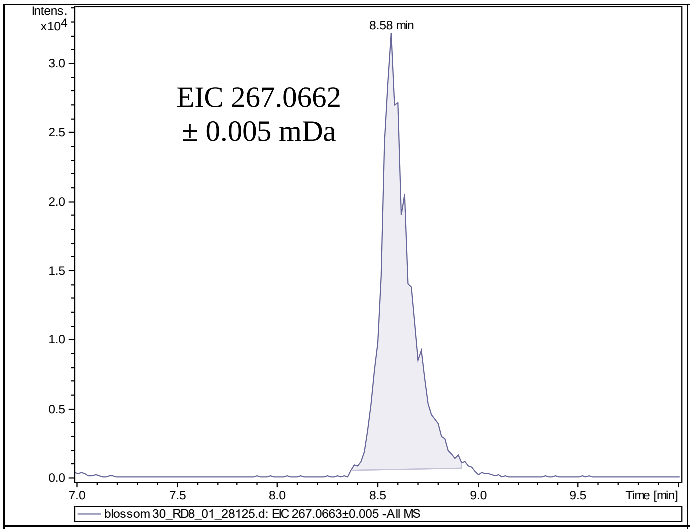

Fig S5a. EIC of m/z 267.0662 in a blossom Honey

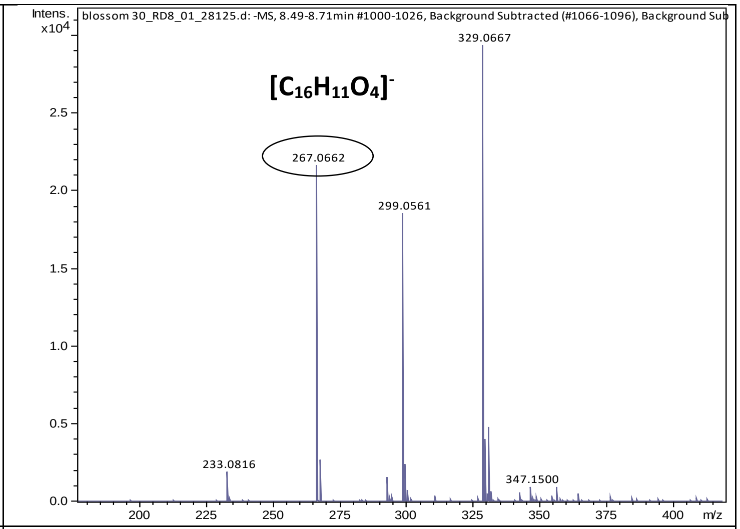

Fig S5b. Background subtracted MS Spectra from 8.5 to 8.7 min

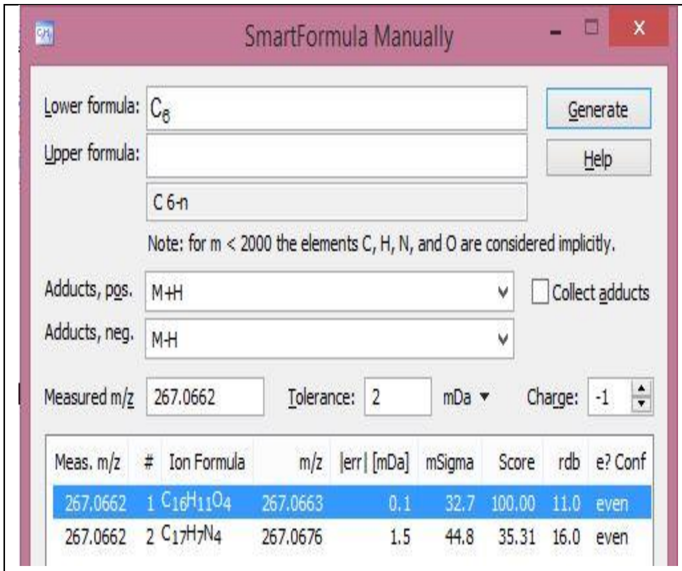

Fig S5c. Molecular Formula Annotation of m/z 267.0662

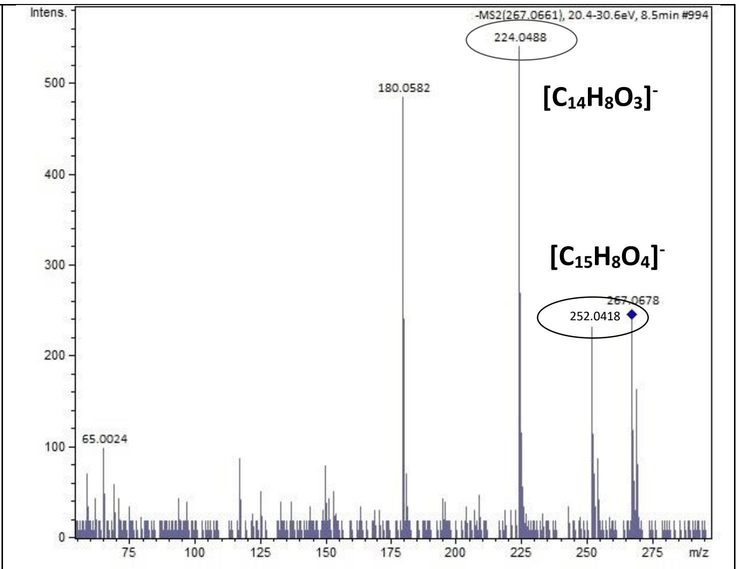

Fig S5d. DDA MS/MS Spectra of 267.0662

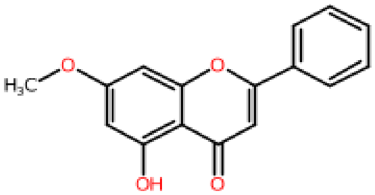

$C_{16}H_{12}O_4$

Tectochrysin

Precursor ion

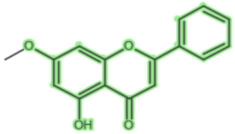

$[C_{15}H_8O_4]^-$

Mass error: 0.3 mDa

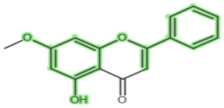

$[C_{14}H_8O_3]^-$

Mass error: 0.3 mDa

Fragment ions

Fig S5e. Structures of precursor and fragment ions of Tectochrysin

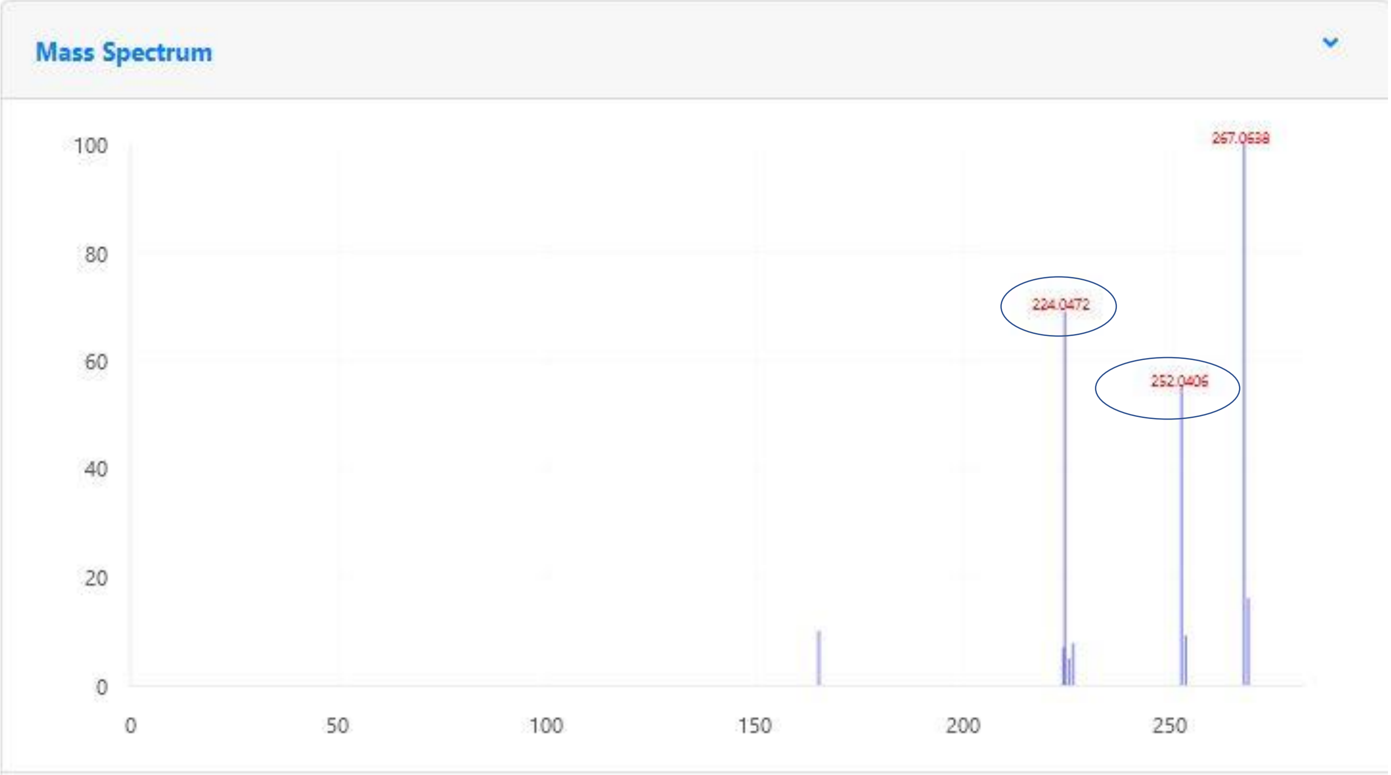

Fig. S5f: Bruker Sumner MetaboBASE Plant Library MetaboBASE0550 (Tectochrysin)

Figure S5. Identification data for the mass feature m/z 299.0561_8.44 min (Tectochrysin)

Supporting information for the non-targeted screening

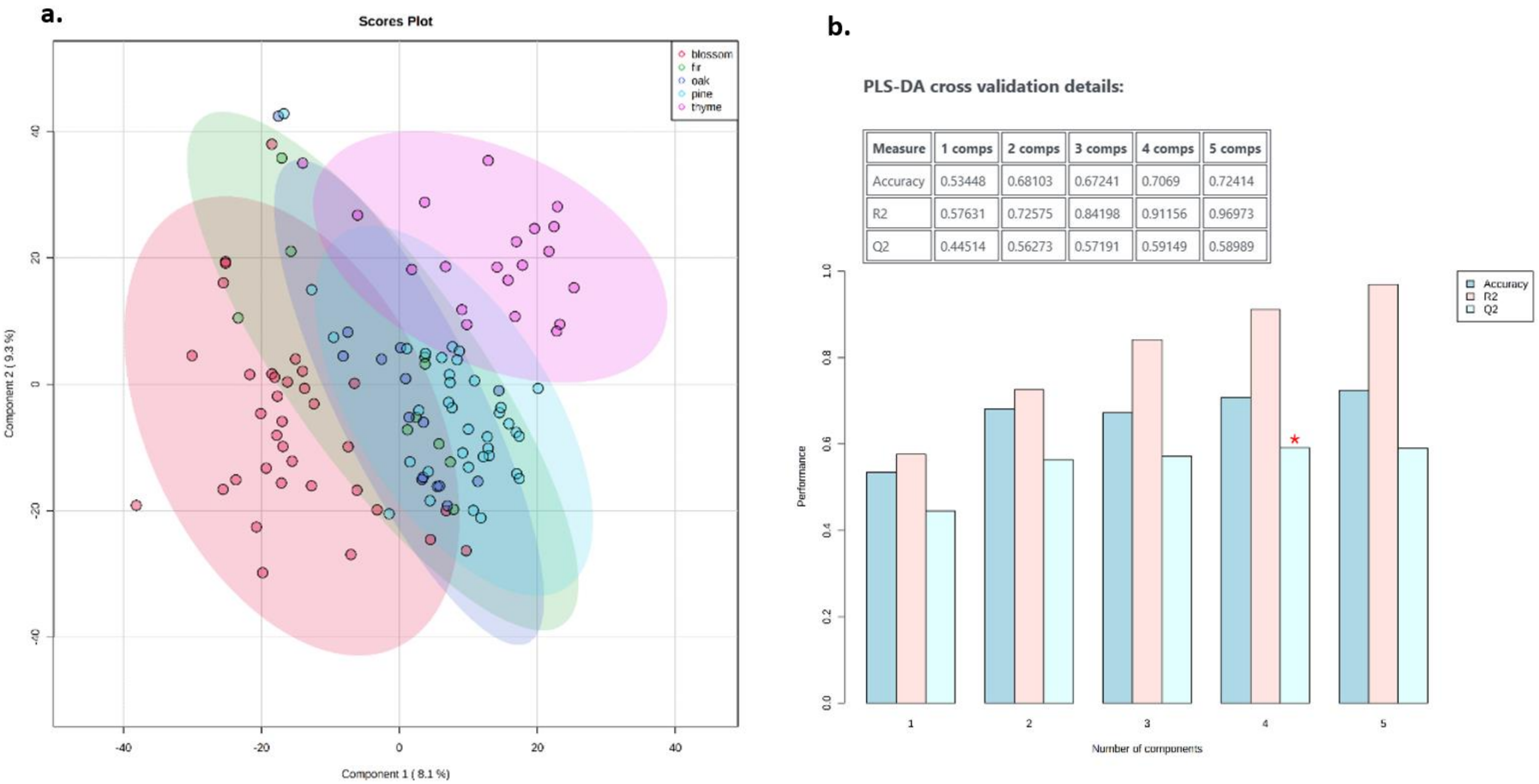

Measure	1 comps	2 comps	3 comps	4 comps	5 comps
Accuracy	0.53448	0.68103	0.67241	0.7069	0.72414
R2	0.57631	0.72575	0.84198	0.91156	0.96973
Q2	0.44514	0.56273	0.57191	0.59149	0.58989

Figure S6. a) The developed PLS-DA model for the 5 different honey classes. b) The effect of the number of parameters in the accuracy, R^2 and Q^2 of the model.

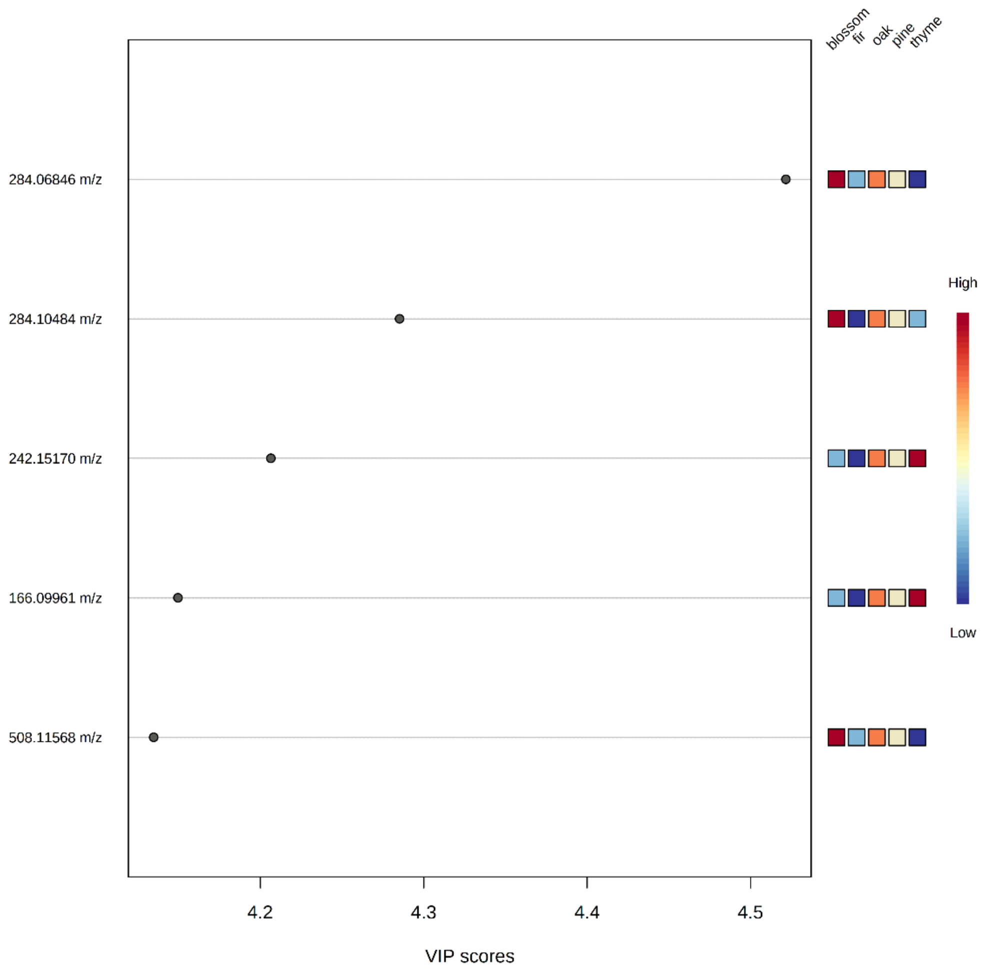

Figure S7. VIP scores on the importance of the monitored mass features for the PLS-DA model containing the 5 honey classes.

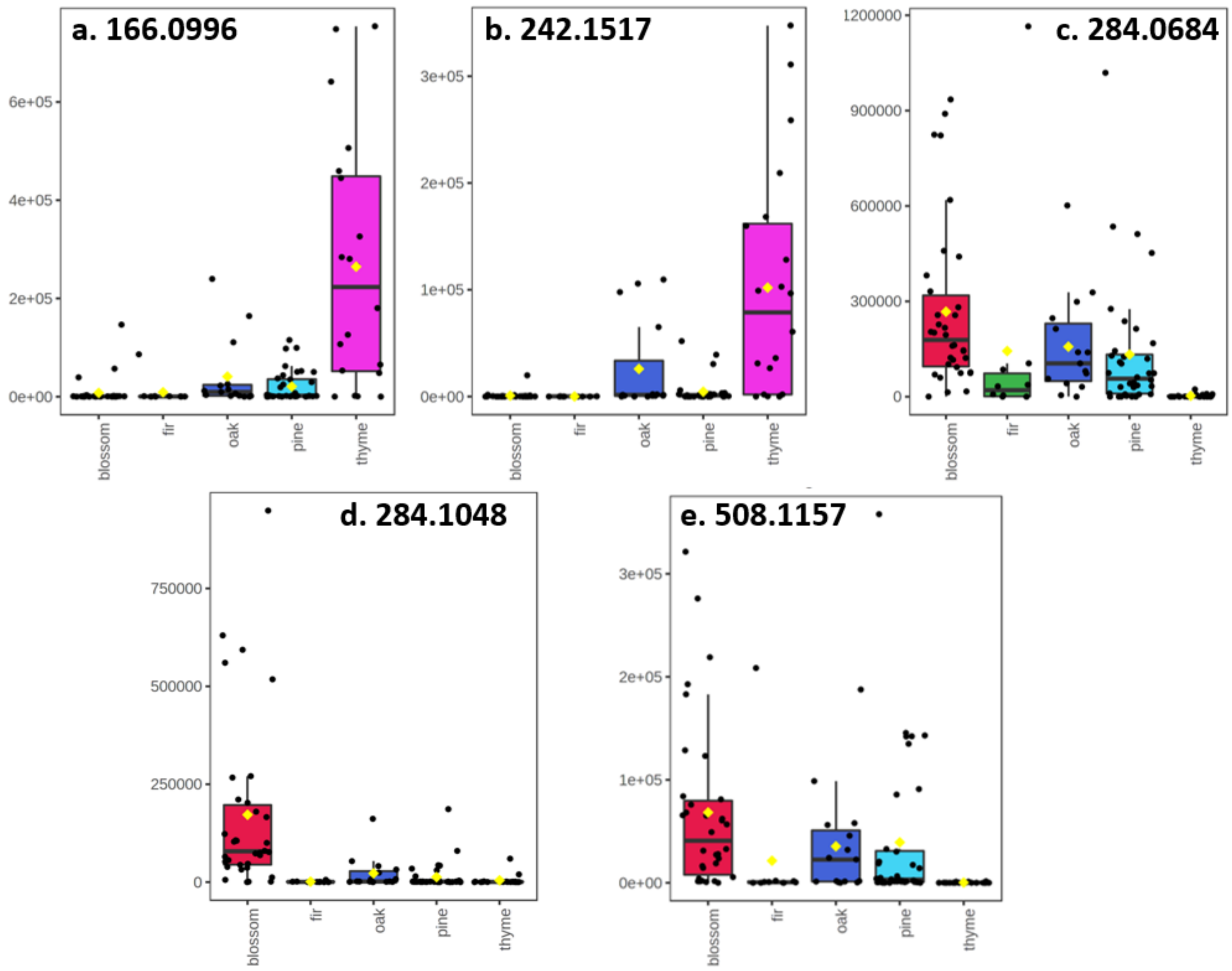

Figure S8. Box plots (Fig. S8a-e) for the 5 VIP mass features for the PLS-DA model containing the 5 honey classes.

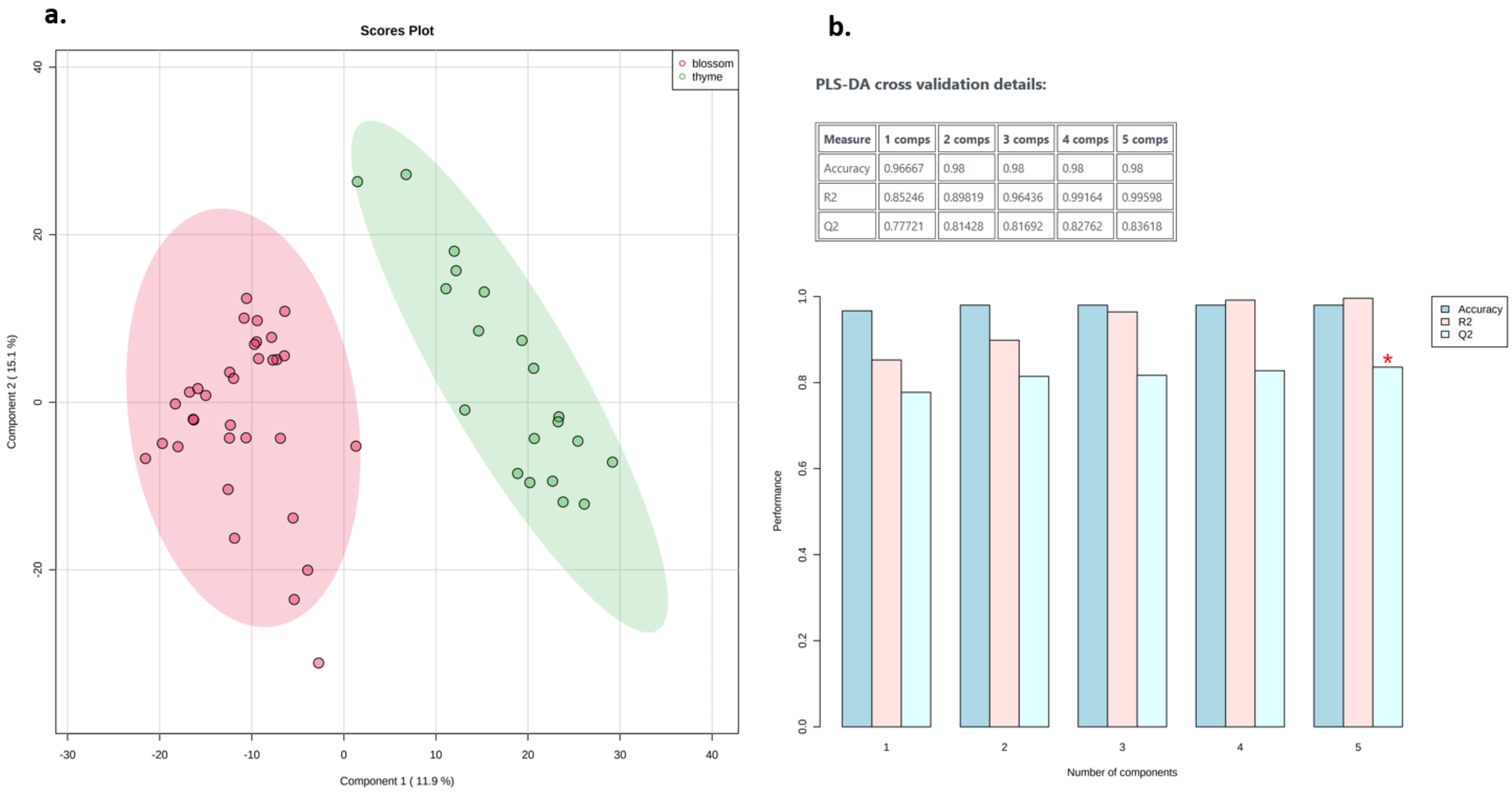

PLS-DA cross validation details:

Measure	1 comps	2 comps	3 comps	4 comps	5 comps
Accuracy	0.96667	0.98	0.98	0.98	0.98
R2	0.85246	0.89819	0.96436	0.99164	0.99598
Q2	0.77721	0.81428	0.81692	0.82762	0.83618

Figure S9. a) The developed PLS-DA model for the binary model “blossom vs. thyme”. b) The effect of the number of parameters in the accuracy, R^2 and Q^2 of the model.

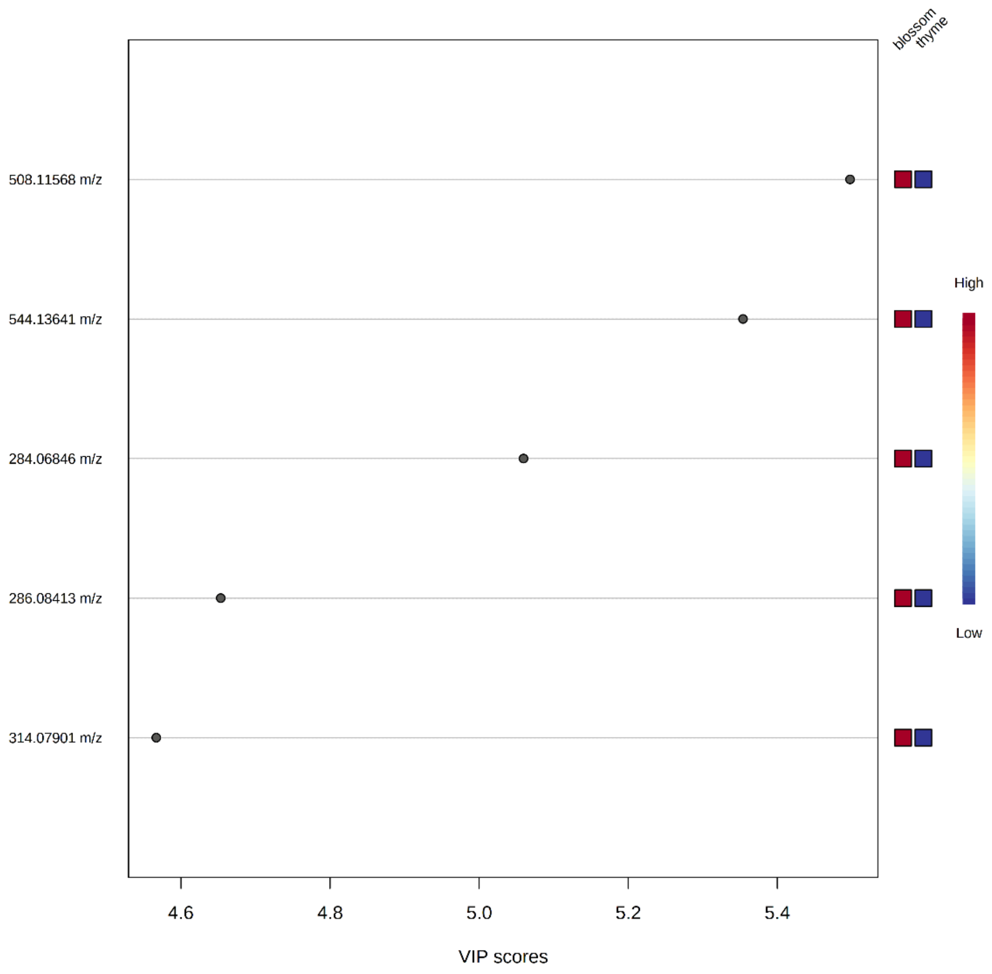

Figure S10. VIP scores on the importance of the monitored mass features for the binary PLS-DA model "blossom vs. thyme".

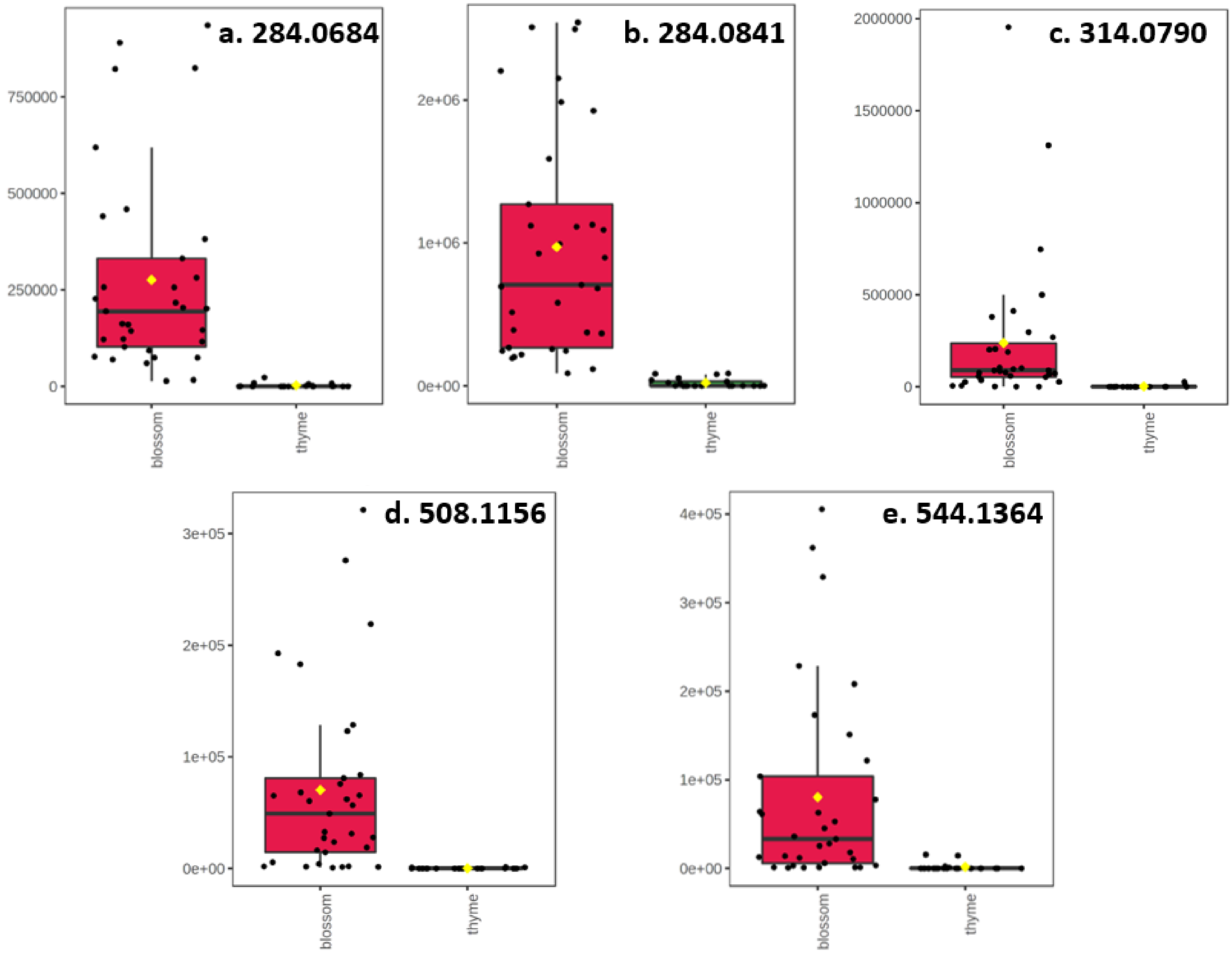

Figure S11. Box plots (Fig. S11a-e) for the 5 VIP mass features for the binary PLS-DA model "blossom vs. thyme".

a.

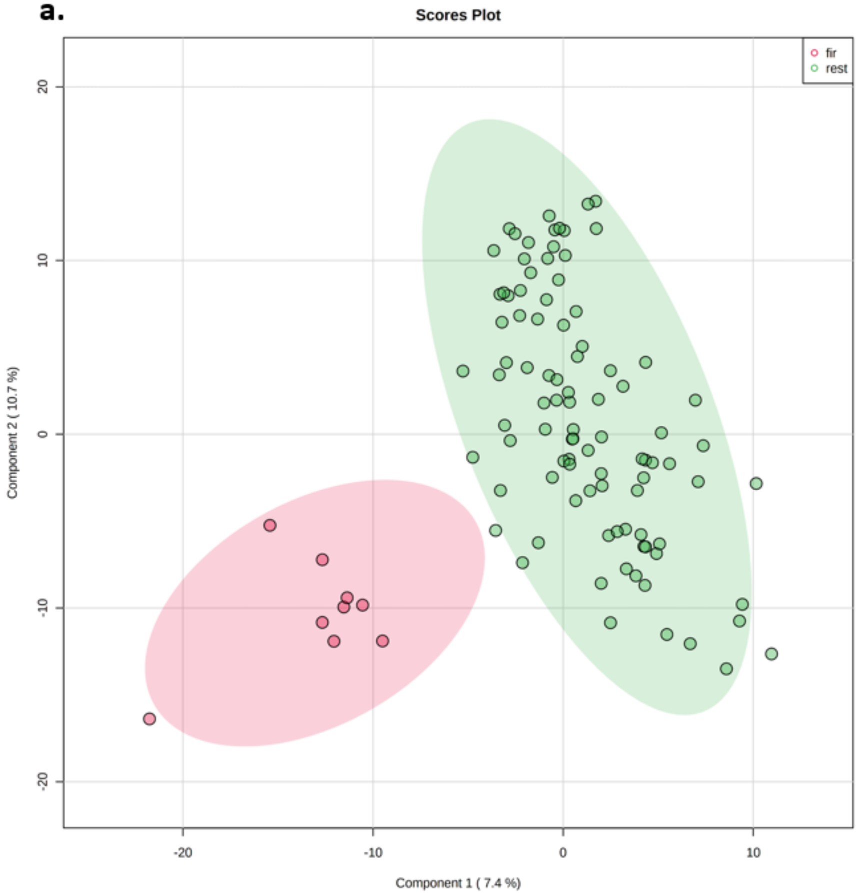

b.

PLS-DA cross validation details:

Measure	1 comps	2 comps	3 comps	4 comps	5 comps
Accuracy	0.93204	0.98058	0.98058	0.99029	0.99029
R2	0.56911	0.75287	0.85315	0.92573	0.95674
Q2	0.39674	0.5669	0.62852	0.63994	0.64662

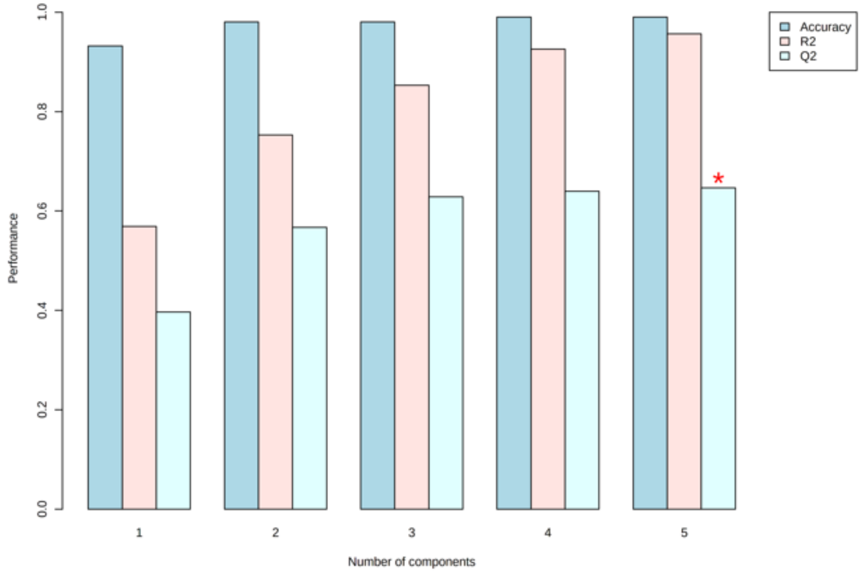

Figure S12. a) The developed PLS-DA model for the binary model “fir vs. rest classes”. b) The effect of the number of parameters in the accuracy, R^2 and Q^2 of the model.

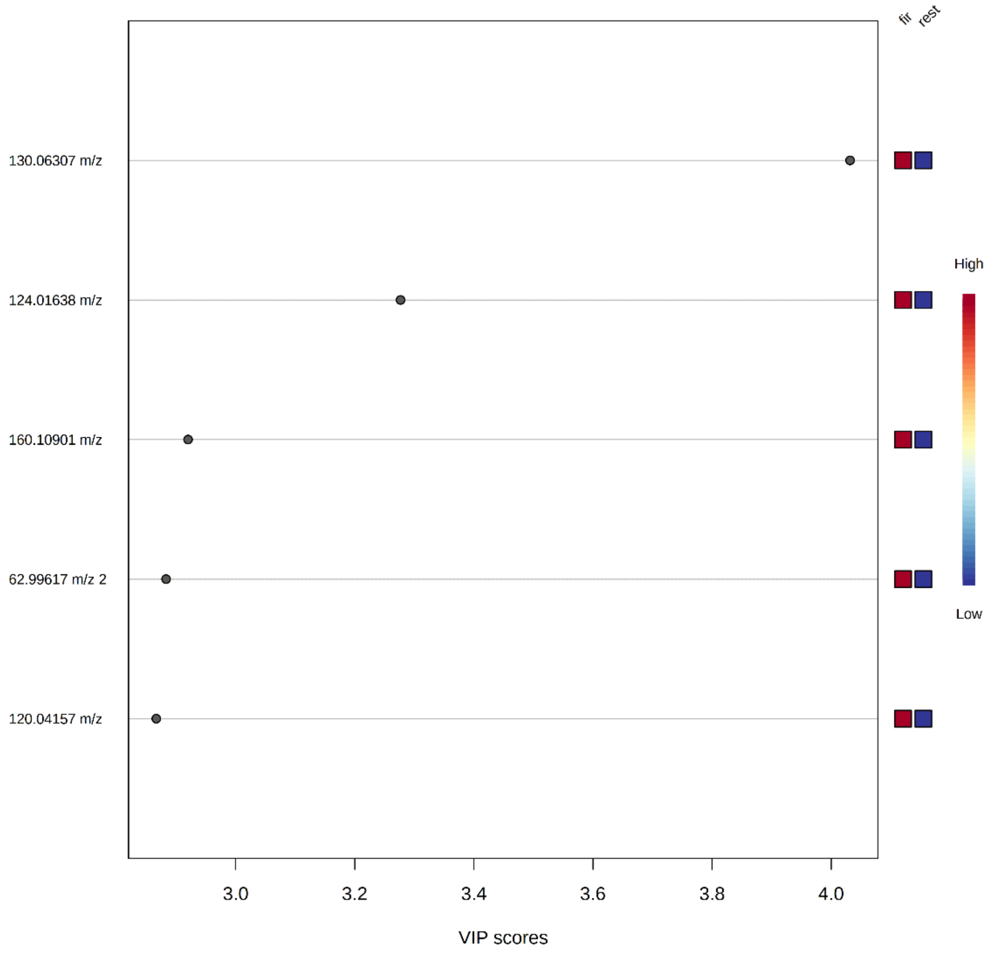

Figure S13. VIP scores on the importance of the monitored mass features for the binary PLS-DA model "fir vs. rest classes".

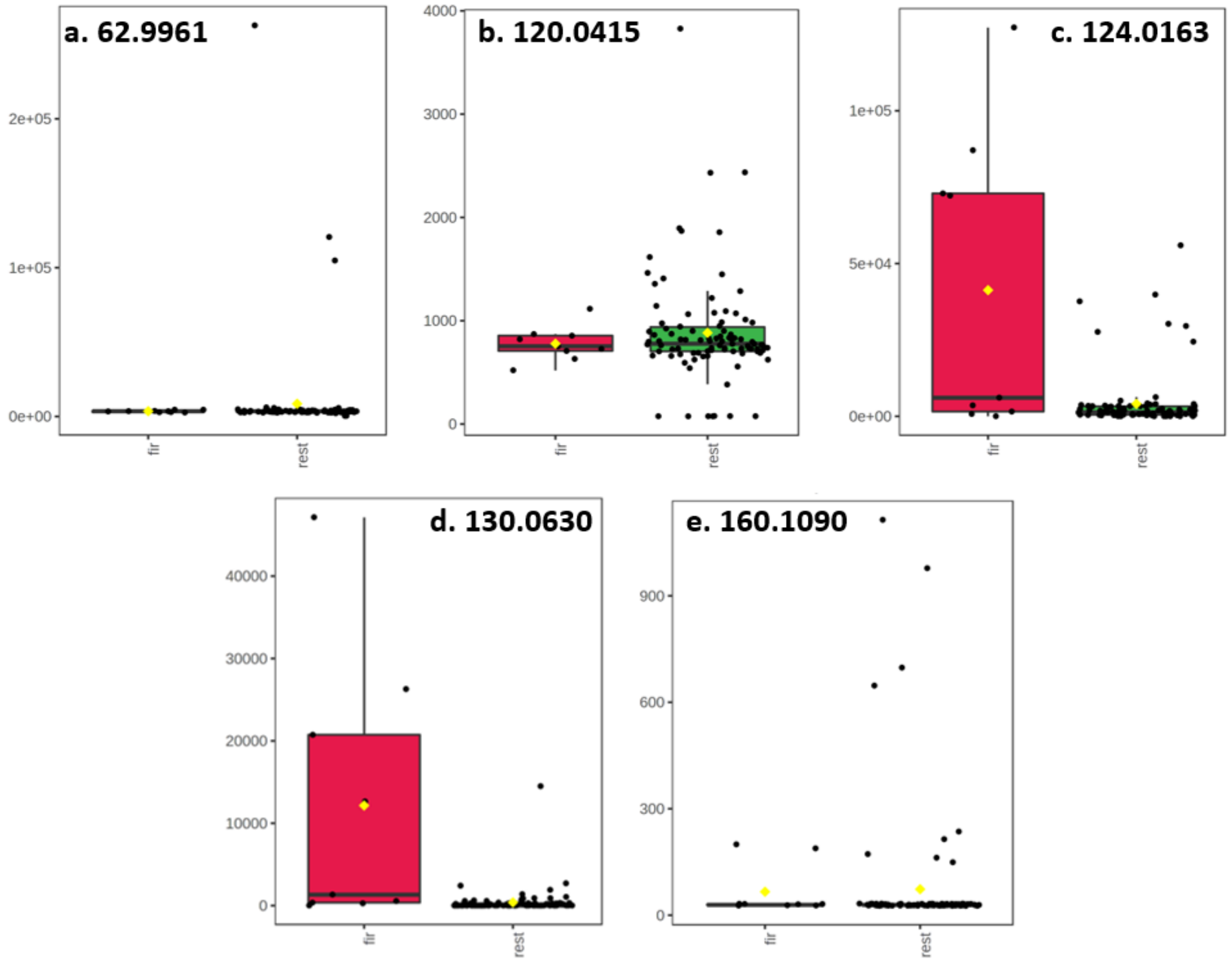

Figure S14. Box plots (Fig. S14a-e) for the 5 VIP mass features for the binary PLS-DA model “fir vs. rest classes”.

a.

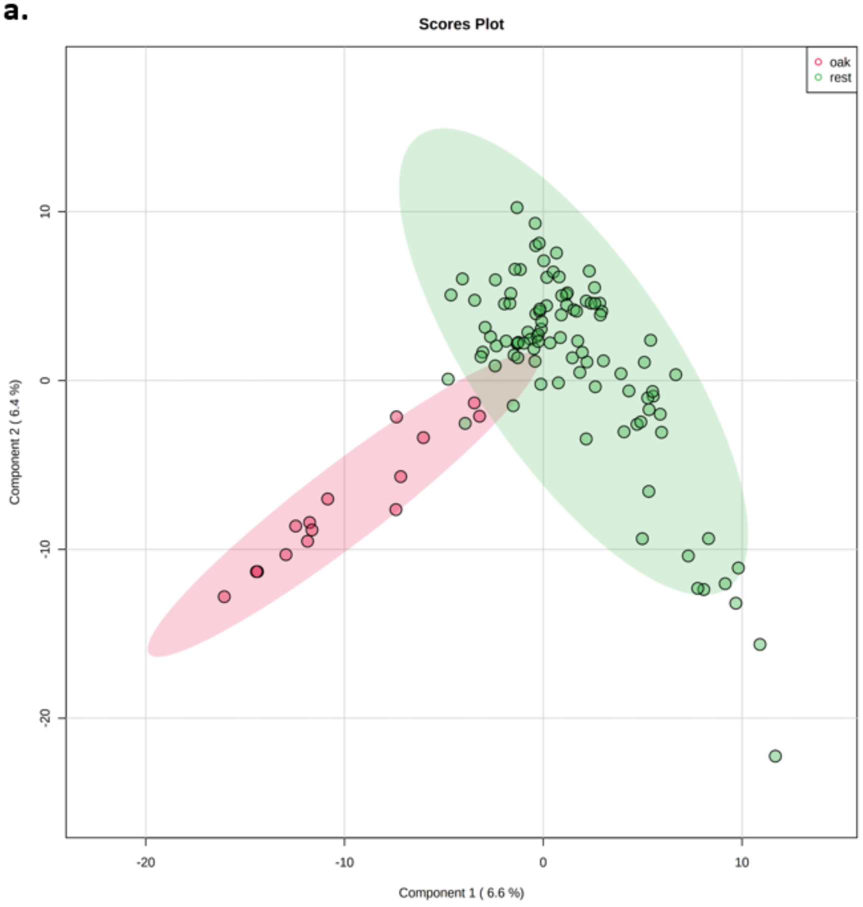

b.

PLS-DA cross validation details:

Measure	1 comps	2 comps	3 comps	4 comps	5 comps
Accuracy	0.944	0.95	0.958	0.968	0.968
R2	0.5407	0.76081	0.8381	0.90504	0.93329
Q2	0.41683	0.609	0.63038	0.62267	0.62411

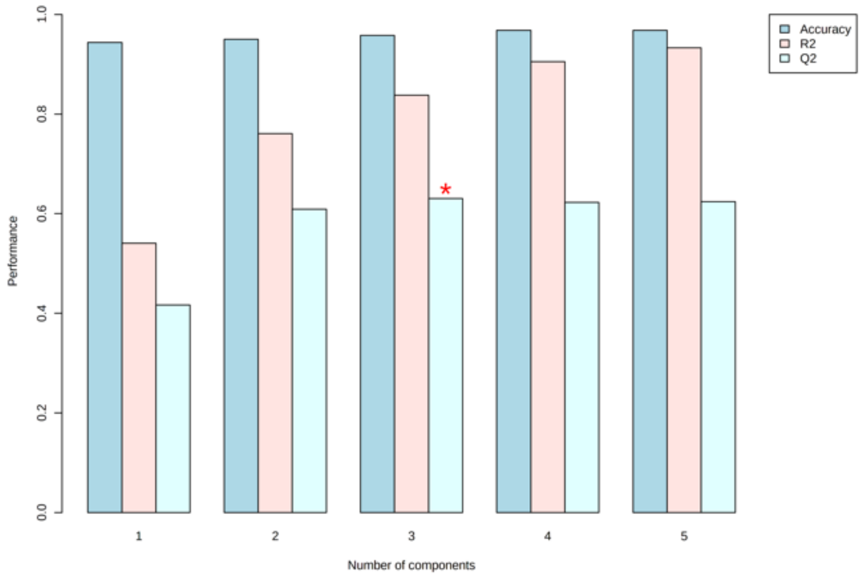

Figure S15. a) The developed PLS-DA model for the binary model “oak vs. rest classes”. b) The effect of the number of parameters in the accuracy, R^2 and Q^2 of the model.

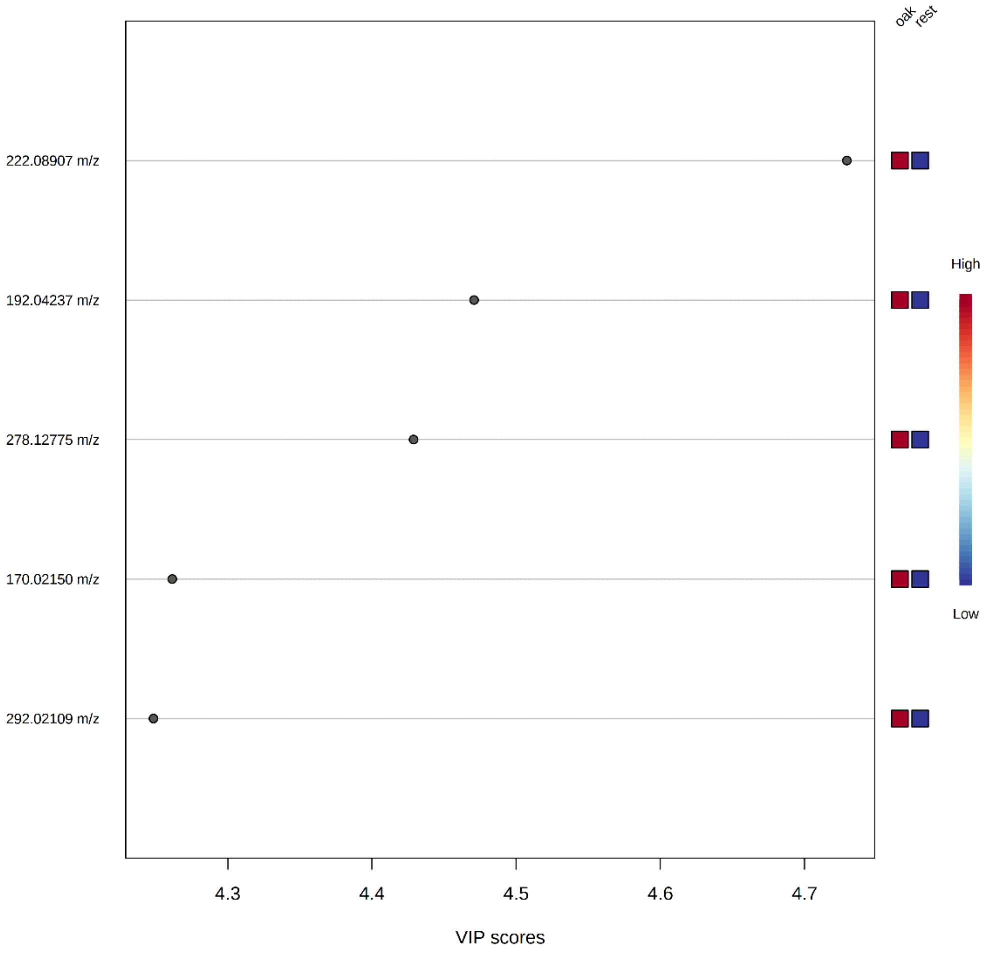

Figure S16. VIP scores on the importance of the monitored mass features for the binary PLS-DA model “fir vs. rest classes”.

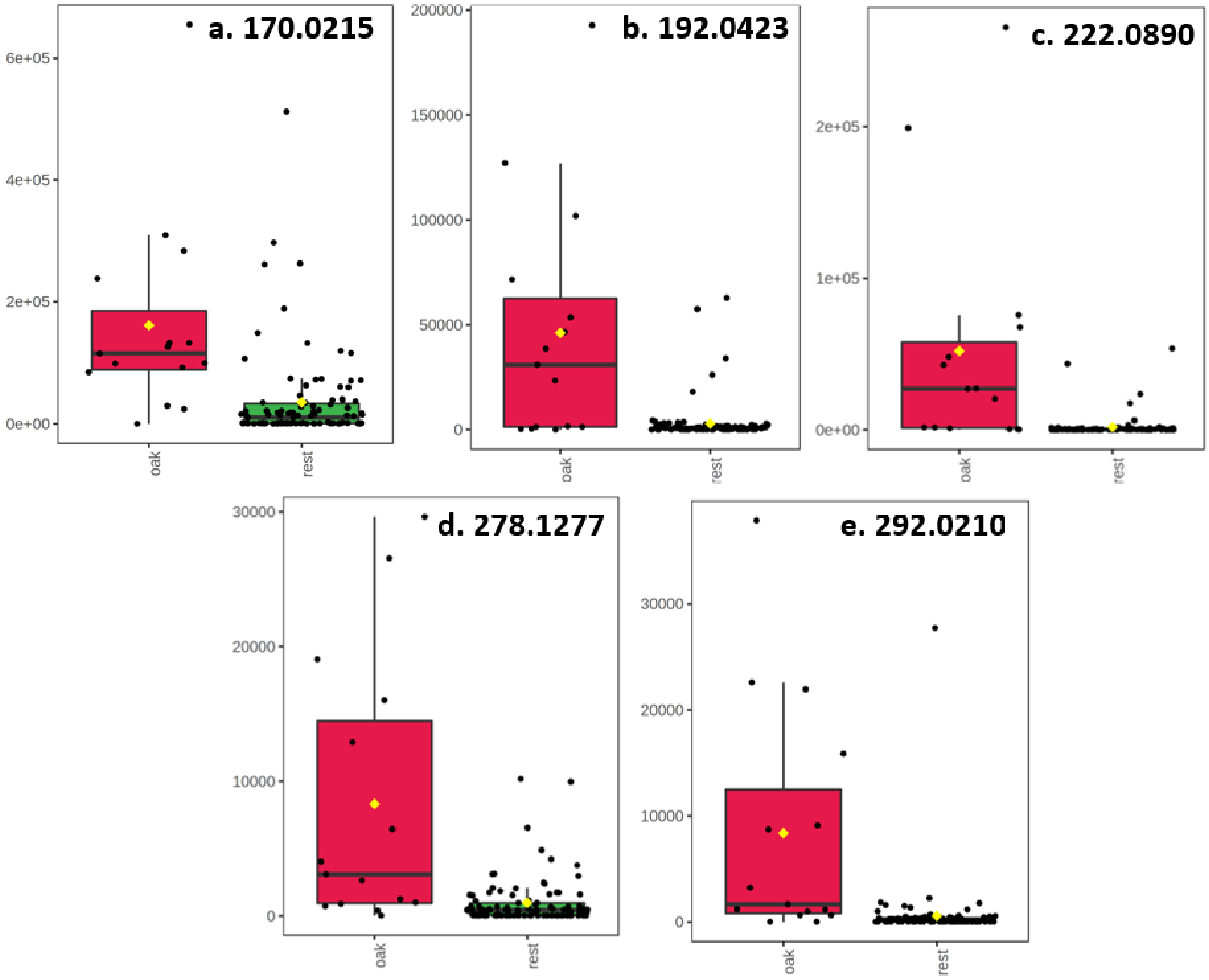

Figure S17. Box plots (Fig. S17a-e) for the 5 VIP mass features for the binary PLS-DA model "oak vs. rest classes".

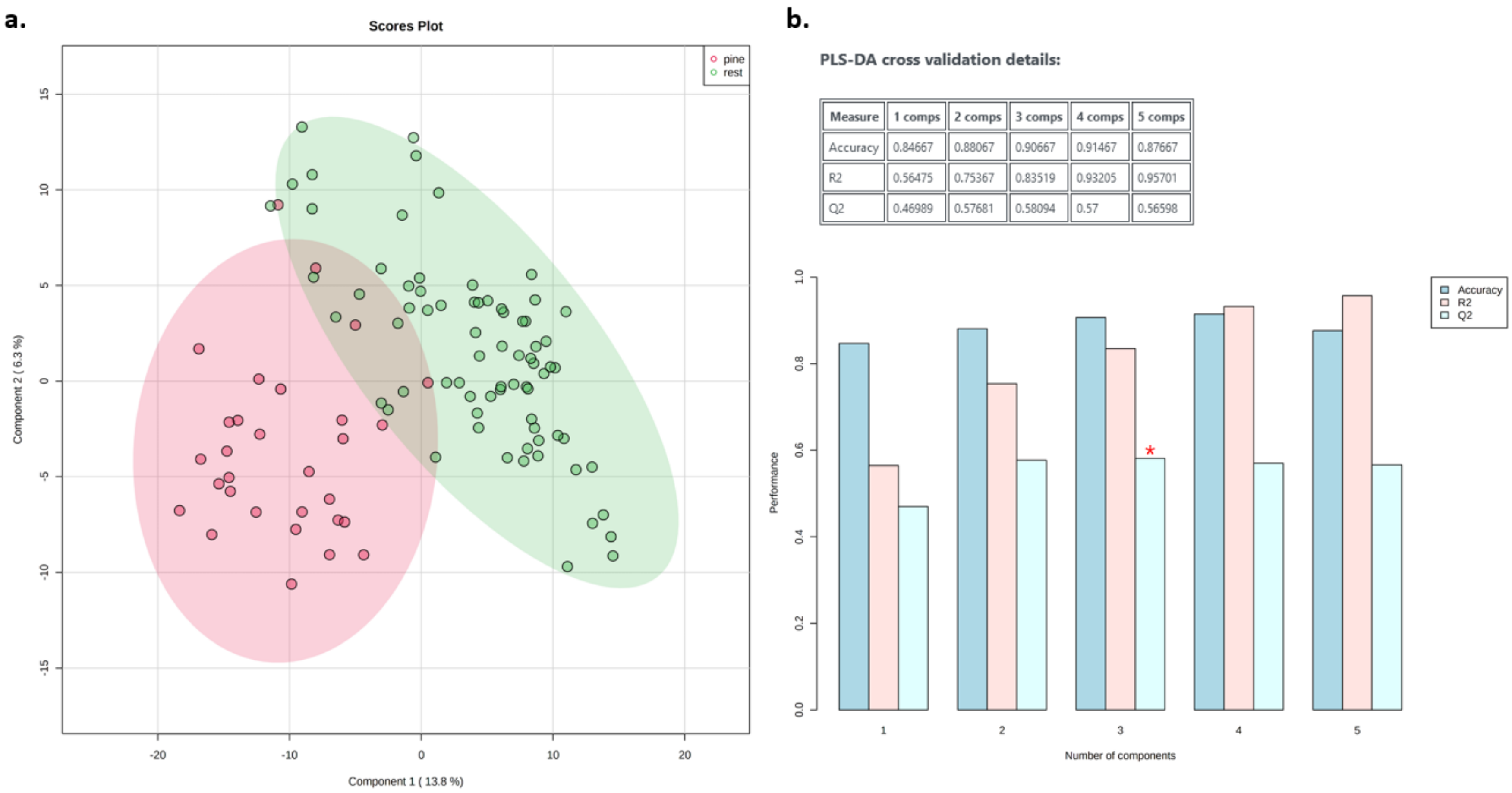

Measure	1 comps	2 comps	3 comps	4 comps	5 comps
Accuracy	0.84667	0.88067	0.90667	0.91467	0.87667
R2	0.56475	0.75367	0.83519	0.93205	0.95701
Q2	0.46989	0.57681	0.58094	0.57	0.56598

Figure S18. a) The developed PLS-DA model for the binary model "pine vs. rest classes". b) The effect of the number of parameters in the accuracy, R^2 and Q^2 of the model.

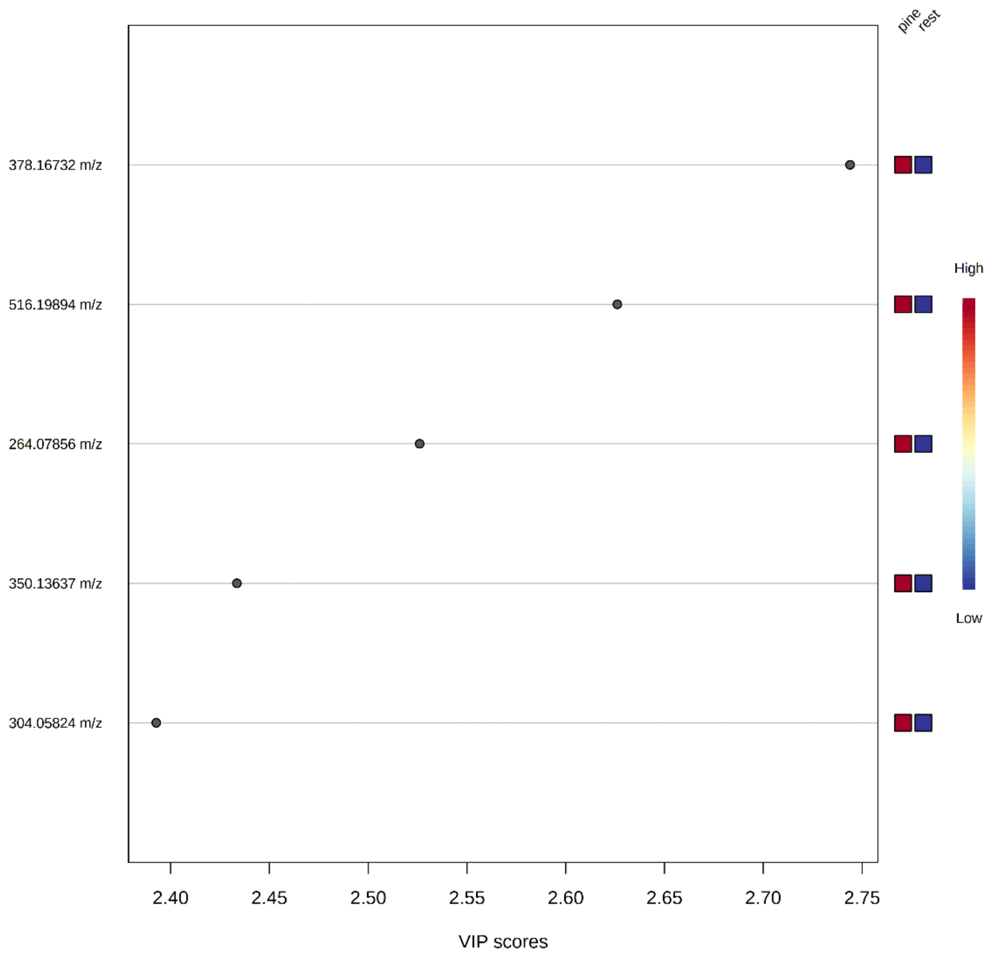

Figure S19. VIP scores on the importance of the monitored mass features for the binary PLS-DA model "pine vs. rest classes".

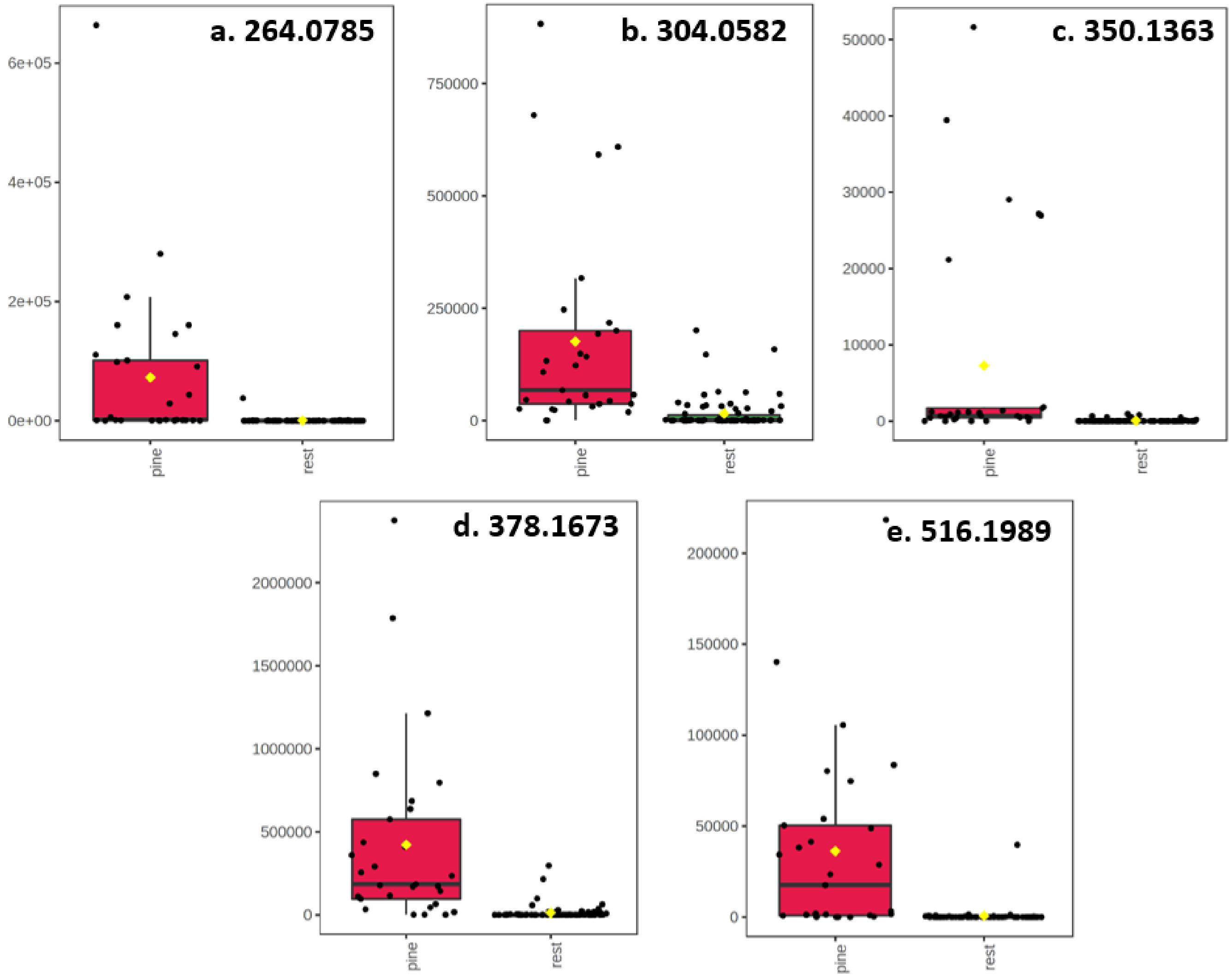

Figure S20. Box plots (Fig. S20a-e) for the 5 VIP mass features for the binary PLS-DA model "pine vs. rest classes".

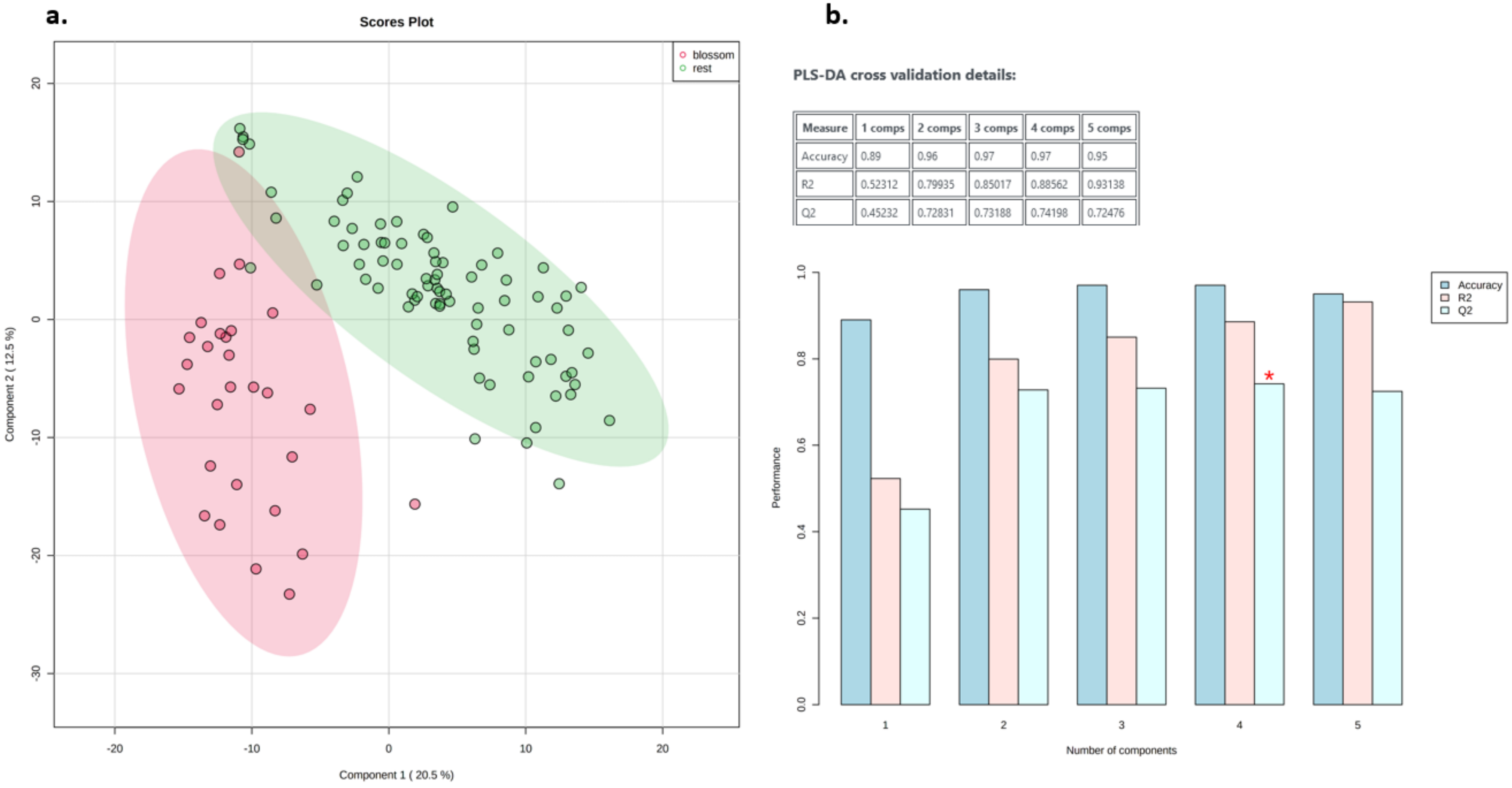

Measure	1 comps	2 comps	3 comps	4 comps	5 comps
Accuracy	0.89	0.96	0.97	0.97	0.95
R2	0.52312	0.79935	0.85017	0.88562	0.93138
Q2	0.45232	0.72831	0.73188	0.74198	0.72476

Figure S21. a) The developed PLS-DA model for the binary model “blossom vs. rest classes”. b) The effect of the number of parameters in the accuracy, R^2 and Q^2 of the model.

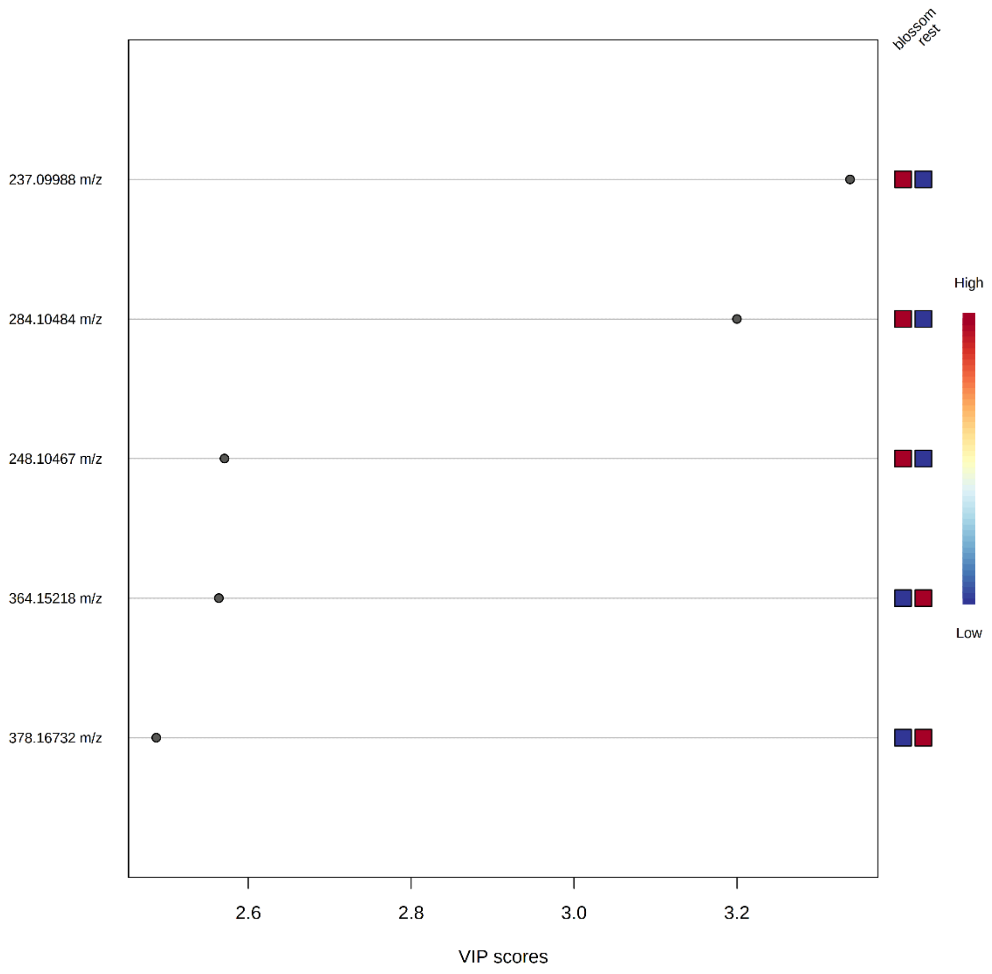

Figure S22. VIP scores on the importance of the monitored mass features for the binary PLS-DA model “blossom vs. rest classes”.

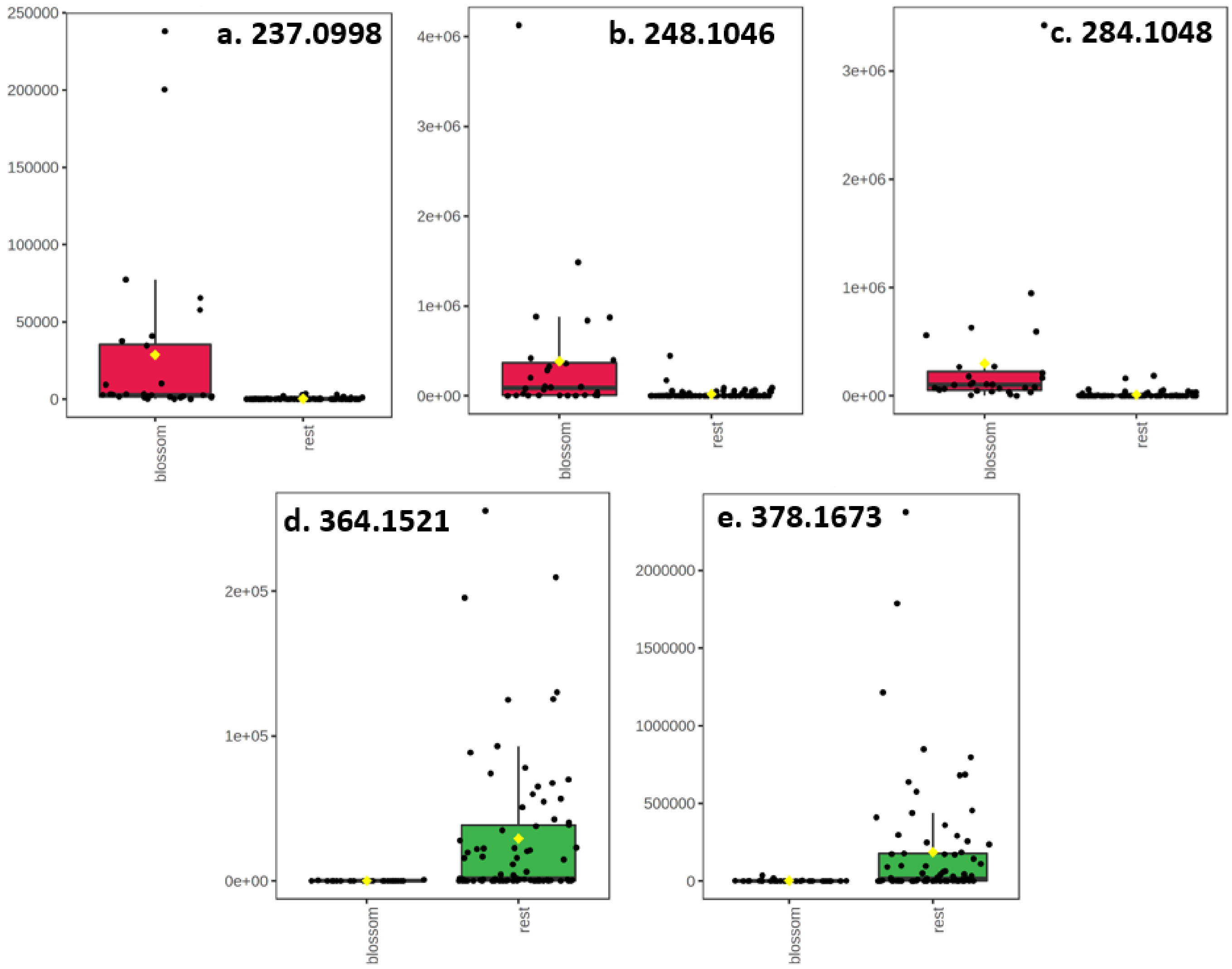

Figure S23. Box plots (Fig. S23a-e) for the 5 VIP mass features for the binary PLS-DA model “blossom vs. rest classes”.

a.

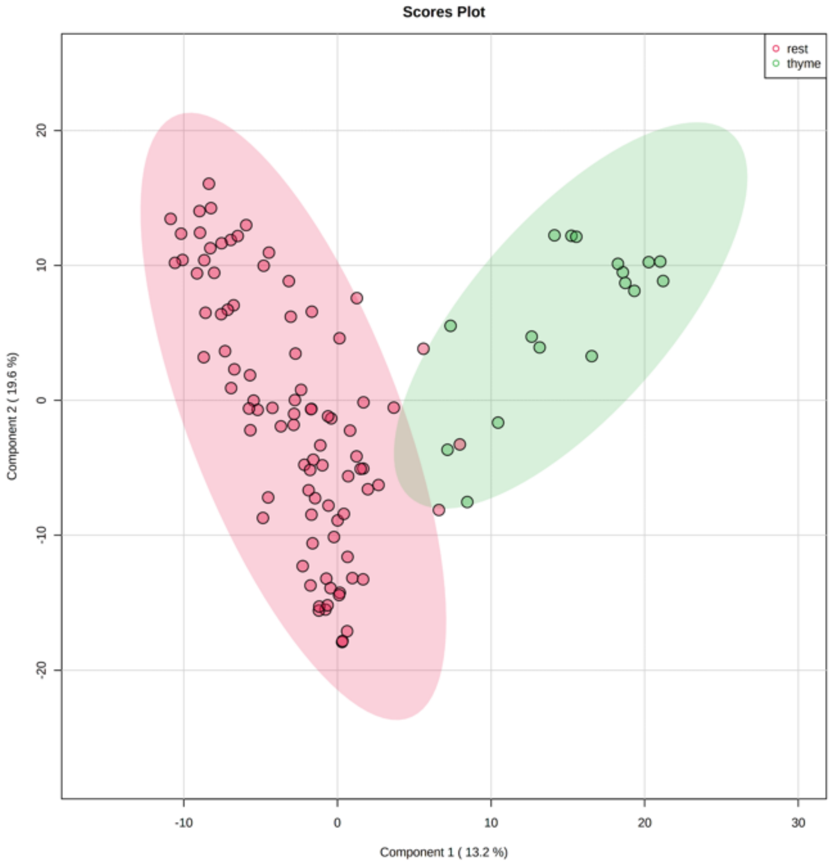

b.

PLS-DA cross validation details:

Measure	1 comps	2 comps	3 comps	4 comps	5 comps
Accuracy	0.972	0.972	0.982	0.982	0.99
R2	0.71866	0.80882	0.8784	0.93209	0.96361
Q2	0.66029	0.75568	0.80527	0.81871	0.82468

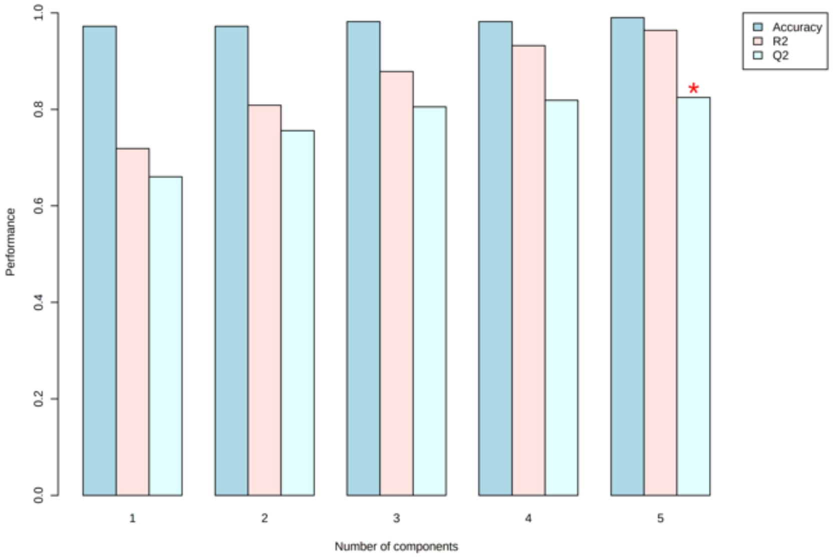

Figure S24. a) The developed PLS-DA model for the binary model “thyme vs. rest classes”. b) The effect of the number of parameters in the accuracy, R^2 and Q^2 of the model.

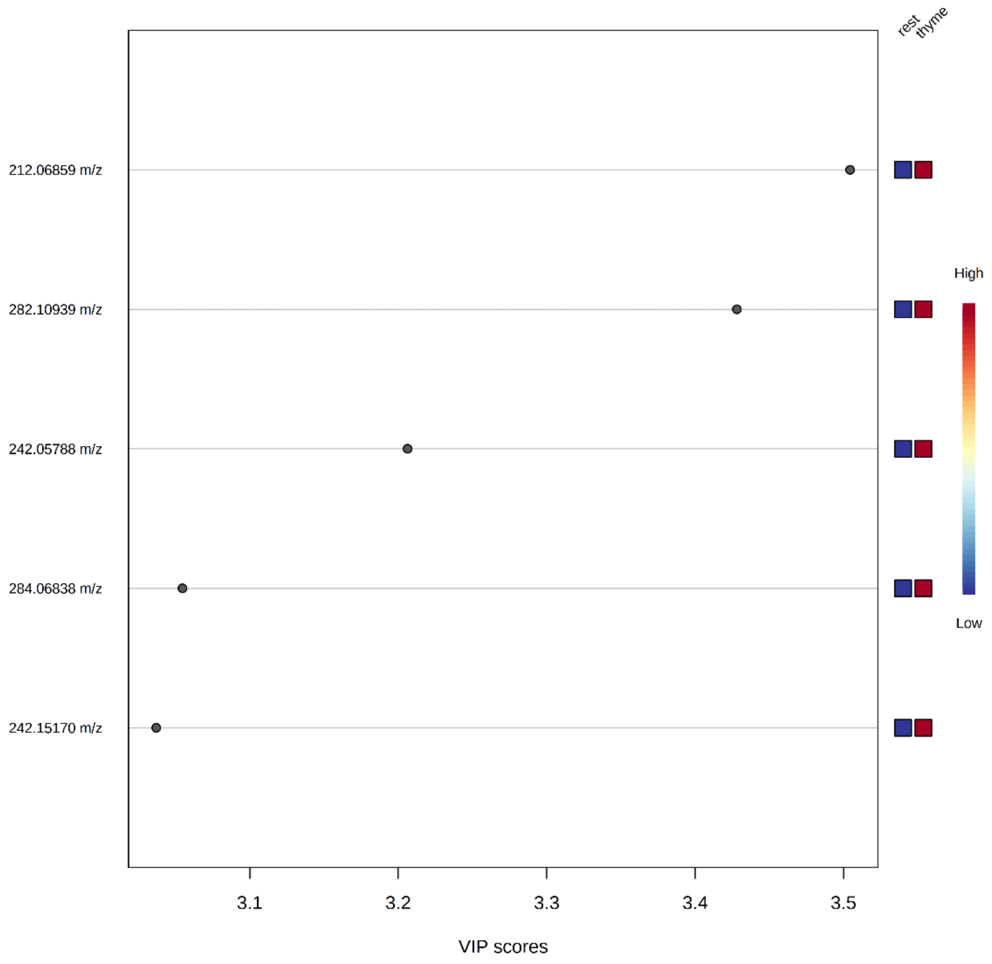

Figure S25. VIP scores on the importance of the monitored mass features for the binary PLS-DA model “thyme vs. rest classes”.

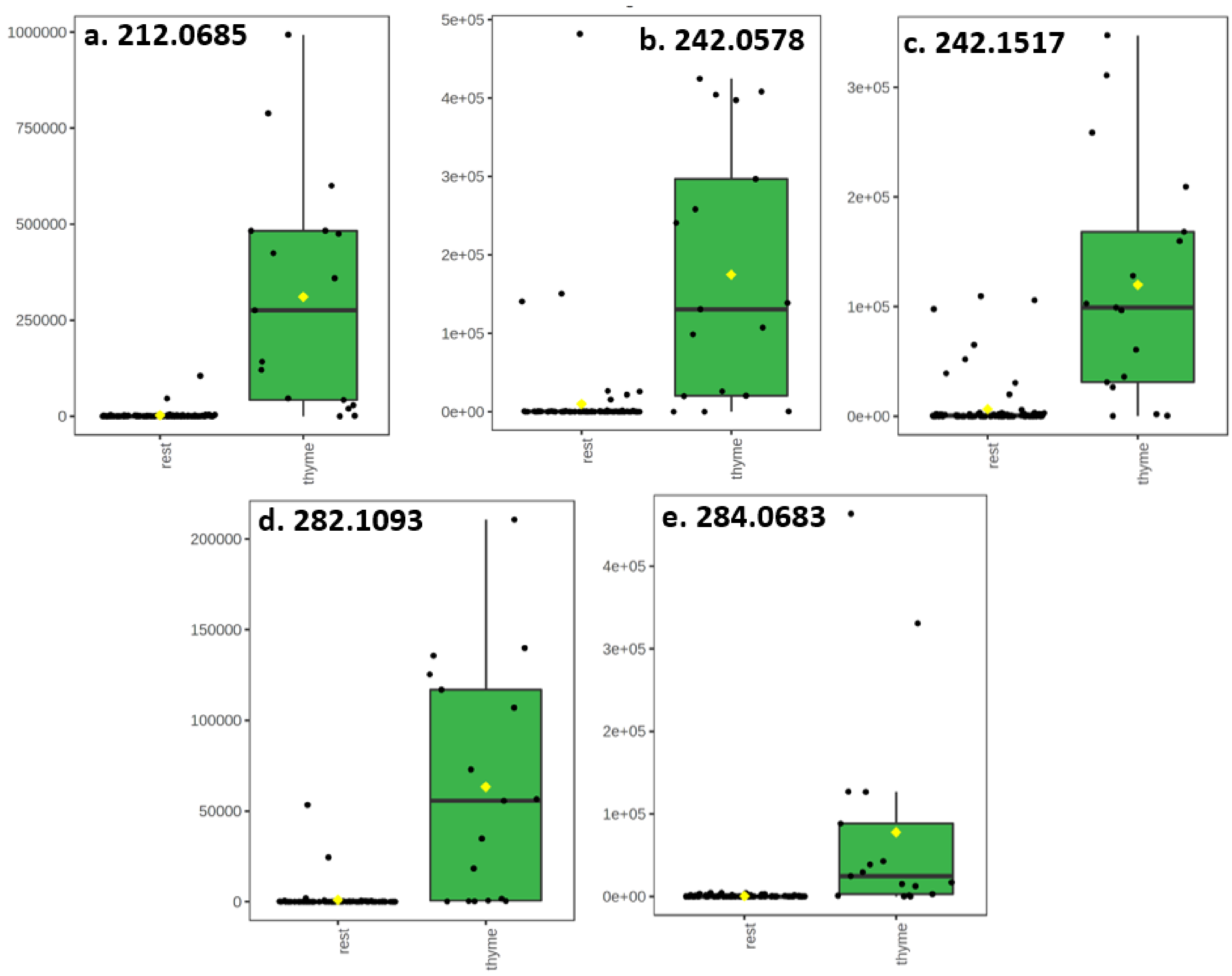

Figure S26. Box plots (Fig. S26a-e) for the 5 VIP mass features for the binary PLS-DA model "thyme vs. rest classes".

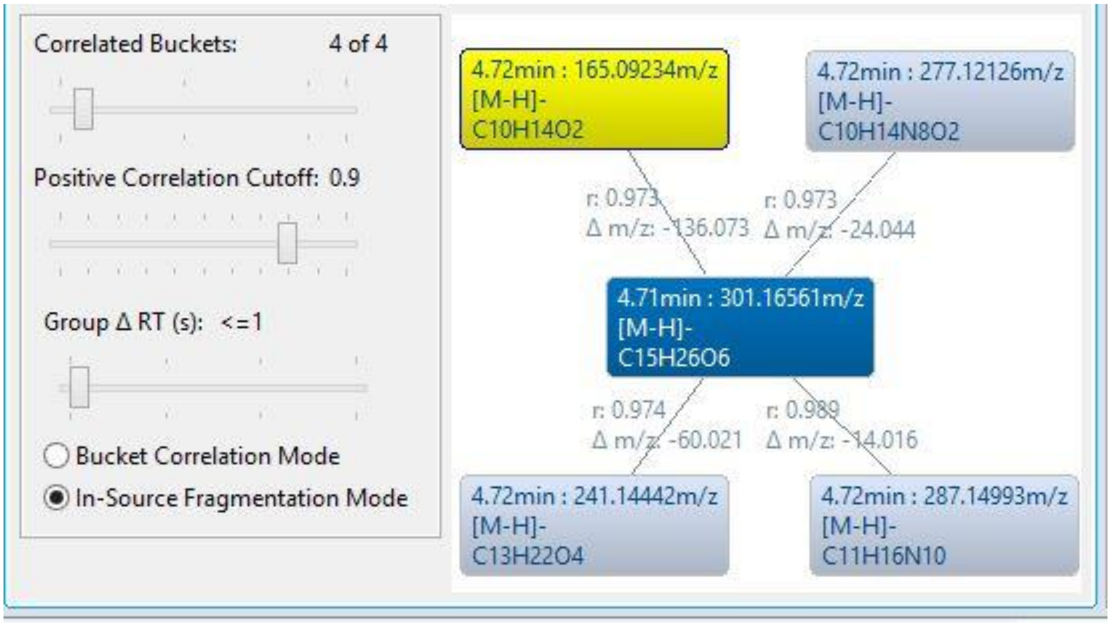

Figure S27. In-source fragments of m/z 301.1656 as proposed by Metaboscape 3.0

Sample list

Table S2. Tested honey sample list

ID	Type	Region	Year
B1	blossom	Evros	2016
B2	blossom	Evros	2016
B3	blossom	Evros	2016
B4	blossom	Evros	2016
B5	blossom	Evros	2016
B6	blossom	Evros	2016
B7	blossom	Evros	2016
B8	blossom	Evros	2016
B9	blossom	Evros	2016
B10	blossom	Macedonia	2016
B11	blossom	Macedonia	2016
B12	blossom	Macedonia	2016
B13	blossom	Macedonia	2016
B14	blossom	Macedonia	2016
B15	blossom	Macedonia	2016
B16	blossom	Macedonia	2016
B17	blossom	Macedonia	2016
B18	blossom	Macedonia	2016
B19	blossom	Macedonia	2016
B20	blossom	Macedonia	2016
B21	blossom	Macedonia	2016
B22	blossom	Macedonia	2016
B23	blossom	Macedonia	2016
B24	blossom	Macedonia	2016
B25	blossom	Macedonia	2016
B26	blossom	Macedonia	2016
B27	blossom	Macedonia	2016
B28	blossom	Macedonia	2016
B29	blossom	Macedonia	2016
B30	blossom	Macedonia	2016
B31	blossom	Macedonia	2016
B32	blossom	Macedonia	2016

B33	blossom	Macedonia	2016
B34	blossom	Macedonia	2016
B35	blossom	Macedonia	2016
B36	blossom	Evros	2017
B37	blossom	Evros	2017
B38	blossom	Evros	2017
B39	blossom	Evros	2017
B40	blossom	Evros	2017
B41	blossom	Evros	2017
B42	blossom	Evros	2017
B43	blossom	Evros	2017
B44	blossom	Evros	2017
B45	blossom	Evros	2017
B46	blossom	Evros	2017
B47	blossom	Evros	2017
B48	blossom	Evros	2017
B49	blossom	Evros	2017
B50	blossom	Evros	2017
B51	blossom	Evros	2017
B52	blossom	Evros	2017
B53	blossom	Evros	2017
B54	blossom	Macedonia	2017
B55	blossom	Macedonia	2017
B56	blossom	Macedonia	2017
B57	blossom	Macedonia	2017
B58	blossom	Macedonia	2017
B59	blossom	Macedonia	2017
B60	blossom	Macedonia	2017
B61	blossom	Macedonia	2017
B62	blossom	Macedonia	2017
O1	oak	n.a.	2016
O2	oak	n.a.	2016
O3	oak	n.a.	2016
O4	oak	n.a.	2016
O5	oak	n.a.	2016
O6	oak	n.a.	2016

O7	oak	n.a.	2016
O8	oak	n.a.	2016
O9	oak	n.a.	2016
O10	oak	n.a.	2016
O11	oak	n.a.	2016
O12	oak	n.a.	2016
O13	oak	n.a.	2016
O14	oak	n.a.	2016
O15	oak	n.a.	2016
O16	oak	n.a.	2017
O17	oak	n.a.	2017
O18	oak	n.a.	2017
O19	oak	n.a.	2017
O20	oak	n.a.	2017
O21	oak	n.a.	2017
O22	oak	n.a.	2017
O23	oak	n.a.	2017
O24	oak	n.a.	2017
F1	fir	Karpenisi	2016
F2	fir	Karpenisi	2016
F3	fir	Karpenisi	2016
F4	fir	Karpenisi	2016
F5	fir	Karpenisi	2016
F6	fir	Karpenisi	2016
F7	fir	Karpenisi	2016
F8	fir	Karpenisi	2016
F9	fir	Karpenisi	2016
F10	fir	Karpenisi	2016
P1	pine	Macedonia	2016
P2	pine	Macedonia	2016
P3	pine	Macedonia	2016
P4	pine	Macedonia	2016
P5	pine	Thasos	2016
P6	pine	Thasos	2016
P7	pine	Thasos	2016
P8	pine	Thasos	2016

P9	pine	Thasos	2016
P10	pine	Thasos	2016
P11	pine	Nikiti	2016
P12	pine	Nikiti	2016
P13	pine	Nikiti	2016
P14	pine	Nikiti	2016
P15	pine	Nikiti	2016
P16	pine	Nikiti	2016
P17	pine	Nikiti	2016
P18	pine	Nikiti	2016
P19	pine	Nikiti	2016
P20	pine	Chalkidiki	2016
P21	pine	Chalkidiki	2016
P22	pine	Chalkidiki	2016
P23	pine	Chalkidiki	2016
P24	pine	Chalkidiki	2016
P25	pine	Chalkidiki	2016
P26	pine	Chalkidiki	2016
P27	pine	Evia	2016
P28	pine	Evia	2016
P29	pine	Evia	2016
P30	pine	Evia	2016
P31	pine	Evia	2016
P32	pine	Evia	2016
P33	pine	Evia	2016
P34	pine	Evia	2016
P35	pine	Evia	2016
P36	pine	Evia	2016
P37	pine	Macedonia	2017
P38	pine	Macedonia	2017
P39	pine	Macedonia	2017
T1	thyme	Chania	2016
T2	thyme	Chania	2016
T3	thyme	Chania	2016
T4	thyme	Chania	2016
T5	thyme	Chania	2016

T6	thyme	Chania	2016
T7	thyme	Heraklion	2016
T8	thyme	Heraklion	2016
T9	thyme	Heraklion	2016
T10	thyme	Heraklion	2016
T11	thyme	Heraklion	2016
T12	thyme	Heraklion	2016
T13	thyme	Rethymno	2016
T14	thyme	Almyros Volos	2016
T15	thyme	Almyros Volos	2016
T16	thyme	Astypalaia	2016
T17	thyme	Astypalaia	2016
T18	thyme	Skyros	2016
T19	thyme	Heraklion	2017
T20	thyme	Chania	2017
T21	thyme	Heraklion	2017
T22	thyme	Chania	2017
T23	thyme	Heraklion	2017
T24	thyme	Heraklion	2017
T25	thyme	Heraklion	2017
T26	thyme	Heraklion	2017
T27	thyme	Chania	2017
T28	thyme	Chania	2017
T29	thyme	Rethymno	2017
T30	thyme	Rethymno	2017
T31	thyme	Rethymno	2017
T32	thyme	Heraklion	2017
T33	thyme	Heraklion	2017
T34	thyme	Heraklion	2017

Method verification results

Table S3. Verification results for the targeted analytes

Compound	Repeatability %RSDr (n=6)	Intermediate precision %RSDR (n=18)	LOD (mg kg-1)	LOQ (mg kg-1)	R^2
2,5 dihydroxybenzoic acid	5.0	7.5	0.072	0.21	0.993
3,4 dihydroxybenzoic acid	2.8	4.3	0.083	0.25	0.990
4 hydroxybenzoic acid	3.3	8.2	0.098	0.23	0.980
apigenin	1.8	5.6	0.082	0.24	0.990
caffeic acid	6.2	8.3	0.065	0.19	0.994
chrysin	3.2	7.1	0.032	0.097	0.999
cinnamic acid	5.4	4.6	0.043	0.13	0.997
eriodictyol	3.4	9.6	0.048	0.14	0.997
ferulic acid	3.2	6.6	0.030	0.091	0.999
galangin	3.8	6.9	0.071	0.21	0.993
gallic acid	2.2	9.4	0.067	0.20	0.994
genistein	0.7	8.1	0.081	0.24	0.991
luteolin	11	8.7	0.079	0.24	0.991
naringenin	3.7	11	0.045	0.15	0.997
p-coumaric acid	1.5	7.8	0.16	0.49	0.960
pinobanksin	3.8	11	0.055	0.16	0.996
pinocembrin	6.9	7.9	0.076	0.23	0.992
quercetin	4.5	19	0.067	0.19	0.993
rosmarinic acid	4.5	9.7	0.084	0.25	0.990
salicylic acid	8.9	8.8	0.33	0.99	0.984
syringic acid	4.8	8.5	0.081	0.24	0.991
taxifolin	2.0	6.7	0.084	0.25	0.990
vanillic acid	3.8	4.4	0.12	0.36	0.980
vanillin	3.3	7.7	0.037	0.11	0.998

Method trueness

Table S4. Attained trueness in terms of recovery (R%) per honey matrix.

Sample ID / Analytes	RECOVERIES (%)				
	**Blossom**	**Fir**	**Oak**	**Pine**	**Thyme**
2,5 dihydroxybeznoic acid	55	57	94	84	108
3,4 dihydroxybenzoic acid	83	70	74	71	109
4 hydroxybenzoic acid	69	87	68	104	103
apigenin	44	46	94	59	76
caffeic	96	61	69	64	79
chrysin	96	93	96	80	88
cinnamic acid	101	73	101	71	85
eriodictyol	105	90	94	56	91
ferulic acid	96	72	98	79	74
galangin	60	45	102	56	90
gallic acid	47	45	66	62	66
genistein	93	102	104	102	76
luteolin	41	91	88	57	50
naringenin	59	99	85	68	106
p-coumaric acid	108	62	65	83	82
pinobanksin	74	99	85	72	106
pinocembrin	66	84	106	102	108
quercetin	47	68	100	35	85
rosmarinic acid	72	61	94	82	107
salicylic acid	66	88	56	106	52
syringic acid	62	61	88	87	99
taxifolin	47	48	67	42	52
vanillic acid	86	101	78	101	95
vanillin	95	107	98	84	78

Table S5. Acquired matrix effects (MEs) per honey matrix.

Sample ID / Analytes	Matrix effect (ME%)				
	Blossom	Fir	Oak	Pine	Thyme
2,5 dihydroxybeznoic acid	14	12	28	21	32
3,4 dihydroxybenzoic acid	31	33	20	22	32
4 hydroxybezoic acid	32	31	32	33	33
apigenin	4.0	1.3	9	9	31
caffeic	32	11	28	33	30
chrysin	28	17	26	33	17
cinnamic acid	-7.0	3.8	5.7	12	28
eriodictyol	11	13	-1.5	3.1	11
ferulic acid	6.0	9.1	24	25	34
galangin	-6.8	-2.2	-6.2	-10	-9.3
gallic acid	33	33	34	31	31
genistein	7.9	3.1	13	12	33
hesperitin	11	11	11	17	18
luteolin	2.0	-4.4	-21	-9.3	30
naringenin	32	14	33	32	25
p-coumaric acid	6.1	1.2	33	29	31
pinobanksin	32	13	22	29	22
pinocembrin	19	-3.3	11	22	8.1
quercetin	-9.2	12	-16	-12	6.2
rosmarinic acid	-14	-7.1	-12	-9.1	12
salicylic acid	15	25	32	25	34
syringic acid	8.3	6.0	6.2	7.0	27
taxifolin	24	32	34	32	33
vanillic acid	18	10	19	18	30
vanillin	-1.4	1.0	-1.0	10	34

Table S6. LC gradient elution and flow rate program

Time (min)	Flow rate (mL/min)	%A	%B
0	0.2	99	1
1.0	0.2	99	1
3.0	0.2	61	39
14.0	0.4	0.1	99.9
16.0	0.48	0.1	99.9
16.1	0.48	99	1
19.0	0.48	99	1
19.1	0.2	99	1
20.0	0.2	99	1

Target Screening Database

Table S7. Target list of phenolic compounds

Compound	Molecular formula	[M−H]⁻ m/z	tR (min)	q1 m/z	q1 formula	q2 m/z	q2 formula	q3 m/z	q3 formula
2,5-dihydroxybenzoic acid (gentistic acid)	$C_7H_6O_4$	153.0193	1.9	108.0215	$C_6H_4O_2$	109.0278	$C_6H_5O_2$		
3,4- dihydroxybenzoic acid (Protocatechuic acid)	$C_7H_6O_4$	153.0193	1.3	109.0294	$C_6H_5O_2$	108.0218	$C_6H_4O_2$		
4-hydroxybenzoic acid	$C_7H_6O_3$	137.0244	1.4	93.0342	C_6H_5O	65.0398	C_5H_5		
Apigenin	$C_{15}H_{10}O_5$	269.0455	8.2	269.0455	$C_{15}H_9O_5$	117.0340	C_8H_5O	151.0031	$C_7H_3O_4$
Caffeic acid	$C_9H_8O_4$	179.0349	1.4	135.0453	$C_8H_7O_2$	134.0346	$C_8H_6O_2$		
Chrysin	$C_{15}H_{10}O_4$	253.0506	9.8	63.024	C_5H_3	143.0502	$C_{10}H_7O$	209.0608	$C_{14}H_9O_2$
Cinnamic acid	$C_9H_8O_2$	147.0452	4.1	103.0553	C_8H_7	147.0446	$C_9H_7O_2$		
Eriodictyol	$C_{15}H_{12}O_6$	287.0561	6.3	151.0038	$C_7H_3O_4$	135.045	$C_8H_7O_2$		
Ferulic acid	$C_{10}H_{10}O_4$	193.0506	2.7	134.0361	$C_8H_6O_2$	178.026	$C_9H_6O_4$		
Galangin	$C_{15}H_{10}O_5$	269.0455	9.9	213.0557	$C_{13}H_9O_3$	169.0659	$C_{12}H_9O$	143.0502	$C_{10}H_7O$
Gallic acid	$C_7H_6O_5$	169.0142	1.2	125.0244	$C_6H_5O_3$	69.0344	C_4H_5O	97.0295	$C_5H_5O_2$
Genistein	$C_{15}H_{10}O_5$	269.0455	7.3	63.024	C_5H_3	133.0295	$C_8H_5O_2$	159.0452	$C_{10}H_7O_2$
Hesperitin	$C_{16}H_{14}O_6$	301.0718	7.5	164.0115	$C_8H_4O_4$	151.0037	$C_7H_3O_4$	242.0585	$C_{14}H_{10}O_4$
Luteolin	$C_{15}H_{10}O_6$	285.0405	7.4	285.0405	$C_{15}H_9O_6$	133.0287	$C_8H_5O_2$		
Naringenin	$C_{15}H_{12}O_5$	271.0612	7.0	119.0502	C_8H_7O	151.0037	$C_7H_3O_4$	107.0138	$C_6H_3O_2$
p-coumaric acid	$C_9H_8O_3$	163.0401	2.5	119.0502	C_8H_7O	93.0344	C_6H_5O		
Pinobanksin	$C_{15}H_{12}O_5$	271.0612	7.2	253.0495	$C_{15}H_9O_4$	197.0597	$C_{13}H_9O_2$	225.0546	$C_{14}H_9O_3$
Pinocembrin	$C_{15}H_{12}O_4$	255.0663	9.0	151.0037	$C_7H_3O_4$	213.0557	$C_{13}H_9O_3$	107.0139	$C_6H_3O_2$
Quercetin	$C_{15}H_{10}O_7$	301.0354	7.1	151.0036	$C_7H_3O_4$	301.0354	$C_{15}H_9O_7$	121.0288	$C_7H_5O_2$
Rosmarinic acid	$C_{18}H_{16}O_8$	359.0772	4.3	161.0233	$C_9H_5O_3$	197.0444	$C_9H_9O_5$	179.0338	$C_9H_7O_4$
salicylic acid	$C_7H_6O_3$	137.0244	3.2	93.0340	C_6H_5O	65.0399	C_5H_5		
Syringic acid	$C_9H_{10}O_5$	197.0455	1.4	123.0080	$C_6H_3O_3$	166.9976	$C_7H_3O_5$		
Taxifolin	$C_{15}H_{12}O_7$	303.0510	4.7	125.0227	$C_6H_5O_3$	285.0408	$C_{15}H_9O_6$	153.0193	$C_7H_5O_4$
Vanillic acid	$C_8H_8O_4$	167.0350	1.4	125.0244	$C_6H_5O_3$	152.0115	$C_7H_4O_4$		
Vanillin	$C_8H_8O_3$	151.0401	4.6	136.0158	$C_7H_4O_3$	108.0217	$C_6H_4O_2$		
2-cis,4-trans-Abscisic acid	$C_{15}H_{20}O_4$	263.1289	5.0	204.1156	$C_{13}H_{16}O_2$	219.1391	$C_{14}H_{19}O_2$	151.0765	$C_9H_{11}O_2$
sakuranetin	$C_{16}H_{14}O_5$	285.0769	9.40	119.0502	C_8H_7O	165.0193	$C_8H_5O_4$	93.0346	C_6H_5O
acacetin	$C_{16}H_{12}O_5$	283.0612	10.00	268.0377	$C_{15}H_8O_5$	268.0455	$C_{15}H_9O_5$	239.035	$C_{14}H_7O_4$

Suspect Screening Database

Table S8. Suspect list of bioactive compounds encountered in honey

ID	Compound name	Molecular Formula	Predicted tR (min)	Experimental tR (min)	Monoisotopic Mass	$[M]^-$	$[M-H]^-$	$[M-H_2O-H]^-$	$[M+HCOOH-H]^-$	$[M+CH_3COOH-H]^-$	Qual. 1	Qual. 2	Qual. 3	Qual. 4	Qual. 5	Reference
1	Tricetin	$C_{15}H_{10}O_7$	7.61		302.0427	302.0432	301.0354	283.0248	347.0409	361.0565	301.0407	302.0430	303.0484	149.0275		12
2	Quercetin 3-Methyl Ether	$C_{16}H_{12}O_7$	7.07		316.0583	316.0589	315.0510	297.0405	361.0565	375.0722	271.0269	300.0280	255.0342			12
3	Quercetin 3,4-Diglycoside	$C_{27}H_{30}O_{17}$	5.60		626.1483	626.1488	625.1410	607.1305	671.1465	685.1622	463.0887	301.0354	300.0293	625.1392	179.0007	12
4	Quercetin-O-Rhamnoside	$C_{21}H_{20}O_{11}$	7.03		448.1006	448.1011	447.0933	429.0827	493.0988	507.1144	447.0979	301.0380	255.0305	300.0291	284.0323	12
5	Kaempferol	$C_{15}H_{10}O_6$	7.26	8.17	286.0477	286.0483	285.0405	267.0299	331.0459	345.0616	285.0399	159.0378	117.0346	286.0442	151.0034	12
6	Kaempferol 3-Ome	$C_{16}H_{12}O_6$	7.48		300.0634	300.0639	299.0561	281.0455	345.0616	359.0772						12
7	Kaempferol-7-O-Rhamnoside	$C_{21}H_{20}O_{10}$	7.51		432.1056	432.1062	431.0984	413.0878	477.1038	491.1195	285.0399	284.0326	431.0991	151.0041		12
8	Kaempferol-3-O-Glycosyl	$C_{21}H_{20}O_{11}$	5.93		448.1006	448.1011	447.0933	429.0827	493.0988	507.1144	447.0979	301.038	255.0305	300.0291	284.0323	12
9	Kaempferol-3-Rhamnoside	$C_{21}H_{20}O_{10}$	7.10		431.0978	431.0984	430.0905	412.0800	476.0960	490.1117						12
10	Kaempferol-7-O-Glycosyl	$C_{21}H_{20}O_{11}$	6.25		448.1006	448.1011	447.0933	429.0827	493.0988	507.1144	447.0931	285.0443	284.0285	150.9993	286.0430	12
11	Myricetin 3-Ome	$C_{16}H_{12}O_8$	6.91		332.0532	332.0538	331.0459	313.0354	377.0514	391.0671						12
12	Isorhamnetin	$C_{16}H_{12}O_7$	7.63	7.96	316.0583	316.0589	315.0510	297.0405	361.0565	375.0722	315.0504	300.0272	151.0038	107.0134	108.0212	12
13	4-Dimethylaminobenzoic Acid	$C_9H_{11}NO_2$	3.21		165.0790	165.0795	164.0717	146.0611	210.0772	224.0928						12
14	Chlorogenic Acid	$C_{16}H_{18}O_9$	3.70		354.0951	354.0956	353.0878	335.0772	399.0933	413.1089	191.0575	353.0866	179.0378	161.0270	87.0068	12
15	Homogentisic Acid	$C_8H_8O_4$	3.29	1.68	168.0423	168.0428	167.0350	149.0244	213.0405	227.0561	123.0441	122.0361	108.0203	167.0342	124.0472	12
16	Methyl Syringate	$C_{10}H_{12}O_5$	5.77	5.95	212.0685	212.0690	211.0612	193.0506	257.0667	271.0823						12
17	Acacetin	$C_{16}H_{12}O_5$	7.99		284.0685	284.0690	283.0612	265.0506	329.0667	343.0823	268.0362	283.0609	269.0339	284.0623	240.0442	12
18	2-trans,4-trans-abscisic acid	$C_{15}H_{20}O_4$	4.44		264.1362	264.1367	263.1289	245.1183	309.1344	323.1500	153.0914	219.1382	204.1150	201.1269	203.1059	12
19	2-cis,4-trans-abscisic acid	$C_{15}H_{20}O_4$	4.44		264.1362	264.1367	263.1289	245.1183	309.1344	323.1500	153.0914	219.1382	204.1150	201.1269	203.1059	12
20	Ellagic Acid	$C_{14}H_6O_8$	4.87		302.0063	302.0068	300.9990	282.9884	347.0045	361.0201	283.9935	300.9970	257.0060	229.0137	201.0191	12
21	DL-p-Hydroxyphenyllactic Acid	$C_9H_{10}O_4$	4.25		182.0579	182.0585	181.0506	163.0401	227.0561	241.0718	163.0392	135.0440	119.0490	134.0370	181.0499	12
22	Phenylacetic Acid	$C_8H_8O_2$	4.28		136.0524	136.0530	135.0452	117.0346	181.0506	195.0663	135.0441	134.0362	107.0489	106.0411	136.0476	12
23	2-Methoxybenzoic Acid	$C_8H_8O_3$	3.05		152.0473	152.0479	151.0401	133.0295	197.0455	211.0612						12
24	Trimethoxybenzoic Acid	$C_{10}H_{12}O_5$	3.41		212.0685	212.0690	211.0612	193.0506	257.0667	271.0823						12
25	4-Methoxyphenylacetic Acid	$C_9H_{10}O3$	4.48		166.0630	166.0635	165.0557	147.0452	211.0612	225.0768						12
26	Quercetin 3,3'-Dimethyl Ether	$C_{17}H_{14}O_7$	7.93		330.0740	330.0745	329.0667	311.0561	375.0722	389.0878						12

ID	Compound name	Molecular Formula	Predicted tR (min)	Experimental tR (min)	Monoisotopic Mass	$[M]^-$	$[M-H]^-$	$[M-H_2O-H]^-$	$[M+HCOOH-H]^-$	$[M+CH_3COOH-H]^-$	Qual. 1	Qual. 2	Qual. 3	Qual. 4	Qual. 5	Reference
27	3-Hydroxybenzoic Acid	$C_7H_6O_3$	3.34		138.0317	138.0322	137.0244	119.0139	183.0299	197.0455	93.0332	137.0233	94.0365	138.0268	56.3217	12
28	Dicaffeoylquinic Acid	$C_{25}H_{24}O_{12}$	7.19		516.1268	516.1273	515.1195	497.1089	561.1250	575.1406						12
29	Prenyl Caffeate	$C_{14}H_{16}O_4$	9.12		248.1049	248.1054	247.0976	229.0870	293.1031	307.1187						12
30	Rhamnetin	$C_{16}H_{12}O_7$	7.89		316.0583	316.0589	315.0510	297.0405	361.0565	375.0722	315.0505	165.0194	121.0294	300.0279	193.0141	12
31	Kaempferide	$C_{16}H_{12}O_6$	7.88		300.0634	300.0639	299.0561	281.0455	345.0616	359.0772	284.0323	299.0555	151.0037	164.0114	300.0624	12
32	Sakuranetin	$C_{16}H_{14}O_5$	8.06	9.25	286.0841	286.0847	285.0768	267.0663	331.0823	345.0980	285.0779	165.0199	119.0506	286.0808	243.0664	12
33	Alpinetin	$C_{16}H_{14}O_4$	7.73		270.0892	270.0898	269.0819	251.0714	315.0874	329.1031						12
34	Pinostrobin	$C_{16}H_{14}O_4$	8.23		270.0892	270.0898	269.0819	251.0714	315.0874	329.1031						12
35	Tectochrysin	$C_{16}H_{12}O_4$	8.22	8.58	268.0736	268.0741	267.0663	249.0557	313.0718	327.0874	252.0408	267.0659	268.0684	224.0472	269.0648	12
36	Dihydroxyflavone	$C_{15}H_{10}O_4$	8.12		254.0579	254.0585	253.0506	235.0401	299.0561	313.0718						12
37	DL-β-Phenyllactic acid	$C_9H_{10}O_3$	3.65	3.13	166.0630	166.0635	165.0557	147.0452	211.0612	225.0768						12
38	Kojic Acid	$C_6H_6O_4$	3.01		142.0266	142.0272	141.0193	123.0088	187.0248	201.0405	141.0187	111.0091	113.0238			12
39	Dehydrovomifoliol	$C_{13}H_{18}O_3$	5.42	5.07	222.1256	222.1261	221.1183	203.1078	267.1238	281.1394						12
40	Benzoic Acid	$C_7H_6O_2$	2.94		122.0368	122.0373	121.0295	103.0189	167.0350	181.0506	121.0304	92.0263	120.0213	93.0348		12
41	Lumichrome	$C_{12}H_{10}N_4O_2$	5.85	6.42	242.0804	242.0809	241.0731	223.0625	287.0786	301.0942						12
42	Rutin	$C_{27}H_{30}O_{16}$	6.29		610.1534	610.1539	609.1461	591.1355	655.1516	669.1672	609.1474	301.0366	300.0319	271.0274	343.0538	12
43	Isoquercetin	$C_{21}H_{20}O_{12}$	5.91		464.0955	464.0960	463.0882	445.0776	509.0937	523.1093	463.0891	301.0363	300.0294	271.0248	151.0037	12
44	Sinapic Acid	$C_{11}H_{12}$O5	3.33		224.0685	224.0690	223.0612	205.0506	269.0667	283.0823	149.0258	193.0166	208.0402	164.0498	223.0607	12
45	p-Methoxybenzoic Acid	$C_8H_8O_3$	2.89		152.0473	152.0479	151.0401	133.0295	197.0455	211.0612	92.0263	136.0121	92.0155	141.7980		12
46	Morin	$C_{15}H_{10}O_7$	7.19		302.0427	302.0432	301.0354	283.0248	347.0409	361.0565	151.0021	271.0255	301.0363	299.0208		12
47	p-Methoxycinnamic Acid	$C_{10}H_{10}O_3$	3.08		178.0630	178.0635	177.0557	159.0452	223.0612	237.0768	177.0552	103.0551	133.0663	131.0510	105.0702	12
48	4-Hydroxyacetophenone	$C_8H_8O_2$	4.36		136.0524	136.0530	135.0452	117.0346	181.0506	195.0663						12
49	3,4-Dimethoxycinnamic Acid	$C_{11}H_{12}O_4$	3.32		208.0736	208.0741	207.0663	189.0557	253.0718	267.0874	207.0657	103.055	163.0763	133.0654		12
50	Fisetin	$C_{15}H_{10}O_6$	6.68		286.0477	286.0483	285.0405	267.0299	331.0459	345.0616	135.0093	285.0405	121.0287	255.0270	163.0029	12
51	Naringin	$C_{27}H_{32}O_{14}$	6.79		580.1792	580.1798	579.1719	561.1614	625.1774	639.1931	271.0641	579.1728	151.0029	459.1191	459.1089	12
52	Hesperidin	$C_{28}H_{34}O_{15}$	6.97		610.1898	610.1903	609.1825	591.1719	655.1880	669.2036	301.0736	609.1862	242.0585	151.0037	286.0483	12
53	Isokaempferide	C16H12O6	7.48	8.44	300.0639	300.0639	299.0566	281.0455	345.0621	359.0778	284.0332	255.0295	227.0346	256.037	228.0393	36
54	Caffeic acid isoprenyl ester	C14H14O4	9.12		246.0892	246.0898	245.0825	227.0714	291.0880	305.1037						22
55	(-)-Epigallocatechin gallate	C22H18O11	10.16		458.0849	458.0855	457.0782	439.0671	503.0837	517.0994	169.0163	305.0692	125.0254	166.9998	170.0198	37

ID	Compound name	Molecular Formula	Predicted tR (min)	Experimental tR (min)	Monoisotopic Mass	$[M]^-$	$[M-H]^-$	$[M-H_2O-H]^-$	$[M+HCOOH-H]^-$	$[M+CH_3COOH-H]^-$	Qual. 1	Qual. 2	Qual. 3	Qual. 4	Qual. 5	Reference
56	4-Hydroxyphenylacetic acid	C8H8O3	3.97		152.0473	152.0479	151.0406	133.0295	197.0461	211.0618	107.0491	65.002	107.0127	151.0392	83.0126	38
57	Arbutin	C12H16O7	5.55		272.0896	272.0901	271.0828	253.0717	317.0883	331.1040	108.0217	109.0295	71.0139	85.0296	113.0244	39
58	Baicalein	C15H10O5	7.81		270.0528	270.0534	269.0461	251.0350	315.0516	329.0673	65.0029	139.0508	181.6102	145.0252	163.0064	40
59	Astragalin	C21H20O11	5.93		448.1006	448.1011	447.0938	429.0827	493.0993	507.1150	447.0934	448.0965	285.0401	284.0324	449.0981	41
60	Kynurenic acid	C10H7NO3	2.81		189.0426	189.0431	188.0358	170.0247	234.0413	248.0570	144.0459	144.0594	102.04853	65.99768	115.04437	22
